# Characterization of GSDME in amphioxus provides insights into the functional evolution of GSDM-mediated pyroptosis

**DOI:** 10.1371/journal.pbio.3002062

**Published:** 2023-05-03

**Authors:** Xinli Wang, Xuxia Wei, Yan Lu, Qinghuan Wang, Rong Fu, Yin Wang, Qin Wang, Xiangyan Wang, Shangwu Chen, Anlong Xu, Shaochun Yuan

**Affiliations:** 1 Guangdong Key Laboratory of Pharmaceutical Functional Genes, Southern Marine Science and Engineering Guangdong Laboratory (Zhuhai), State Key Laboratory of Biocontrol, School of Life Sciences, Sun Yat-sen University, Guangzhou, People’s Republic of China; 2 Laboratory for Marine Biology and Biotechnology, Pilot National Laboratory for Marine Science and Technology, Qingdao, People’s Republic of China; 3 School of Life Sciences, Beijing University of Chinese Medicine, Beijing, People’s Republic of China; National Institute of Biological Sciences, CHINA

## Abstract

Members of the gasdermin (GSDM) family are pore-forming effectors that cause membrane permeabilization and pyroptosis, a lytic proinflammatory type of cell death. To reveal the functional evolution of GSDM-mediated pyroptosis at the transition from invertebrates to vertebrates, we conducted functional characterization of amphioxus GSDME (BbGSDME) and found that it can be cleaved by distinct caspase homologs, yielding the N253 and N304 termini with distinct functions. The N253 fragment binds to cell membrane, triggers pyroptosis, and inhibits bacterial growth, while the N304 performs negative regulation of N253-mediated cell death. Moreover, BbGSDME is associated with bacteria-induced tissue necrosis and transcriptionally regulated by BbIRF1/8 in amphioxus. Interestingly, several amino acids that are evolutionarily conserved were found to be important for the function of both BbGSDME and HsGSDME, shedding new lights on the functional regulation of GSDM-mediated inflammation.

## Abstract 2

Characterization of Gasdermin E-mediated pyroptosis in the basal chordate amphioxus reveals that it is transcriptionally regulated by IRF1/8, involved in bacteria induced tissue damage, and inhibited by caspase inhibitor.

## Introduction

Cytokines of the interleukin-1 (IL-1) family are essential determinants of inflammation. Due to the absence of an amino-terminal secretion signal, the mechanisms underlying their protein secretion from phagocytes have attracted intense attention for more than 30 years [1]. In 2015, 3 independent research groups using genetic screening or quantitative mass spectrometry-based analysis identified the pore-forming protein gasdermin D (GSDMD) as the conduit for IL-1 secretion [2–4]. GSDMD is a substrate of activated caspase-1 (CASP1) or CASP4/5/11, allowing it to release the N-terminus and to form inner diameters of 10 to 14 nm oligomeric pores in the cell membranes [5–8]. In addition to inflammasome-mediated pore formation in macrophages, GSDMD can mediate neutrophil pyroptosis, which is essential in NETosis and antimicrobial NET extrusion, revealing fundamental differences in GSDMD trafficking between neutrophils and macrophages [9,10].

GSDMD belongs to the pore-forming protein family [11]. Humans contain 6 GSDM members, including GSDMA, GSDMB, GSDMC, GSDMD, GSDME, and Pejvakin (PJVK) [11]. In addition to inflammatory responses, important progress in GSDM-mediated cell death in septic shock and autoimmune diseases, especially in tumor growth, have been made [12]. GSDME, described as a genetic cause of hearing loss, has been considered as a tumor suppressor. The expression of GSDME is suppressed in many cancers, and such repression could enhance tumor growth but decrease survival [13]. When cleaved by CASP3, GSDME can switch noninflammatory apoptosis to pyroptosis in cancer cells [14]. GSDME can be also cleaved by granzyme B, therefore enhancing the phagocytosis of tumor cells by tumor-associated macrophages, as well as tumor-infiltrating natural killer and CD8^+^ T lymphocytes [15]. GSDMC, transcriptionally enhanced by nucleic PD-L1 in cancer cells, is a substrate of CASP8, leading to tumor necrosis and poor survival [16]. Similar to GSDME, GSDMB is cleaved by granzyme A to unleash its pore-forming activity, resulting in the killing of GSDMB-positive cancer cells through pyroptosis [17]. In addition to targeting the membrane of cancer cells, a new study showed that GSDMB exhibits direct microbiocidal activity through recognition of phospholipids found on gram-negative bacterial membranes, placing GSDMB as a center executioner of intracellular bacterial killing [18]. Recently, GSDMA was reported to trigger pyroptosis in keratinocytes after being cleaved by *streptococcal pyrogenic* exotoxin B (SpeB) in the defense against skin microbial pathogen [19].

After functional characterization of the GSDM family in mammals, GSDM-mediated pyroptosis has been revealed in teleost fish, cnidaria, and even fungi and bacteria. *Danio rerio* (Dr) has 3 GSDM members, the DrGSDMEa, DrGSDMEb, and DrPJVK [14]. Similar to mammalian GSDMD, DrGSDMEb is cleaved by inflammatory CASPs to release its N-terminus to mediate pyroptosis, which is responsible for lethal LPS-induced septic shock and NETosis for bacterial clearance in vivo [20,21]. Different to DrGSDMEs, turbot *Scophthalmus maximus* GSDMEa is cleaved by CASP3/6/7 to generate 2 N-fragments with different functions, while turbot GSDMEb can be cleaved by CASP8 and its NT fragment can not trigger pyroptosis in HEK293T cells [22]. GSDME homologs in coral or another teleost fish *Cynoglossus semibreves* (tongue sole) can be also cleaved by CASPs to exert pyroptotic and bactericidal activities through its N-terminal domain [23,24]. Notably, studies have shown that the regulator cell death (Rcd-1) in fungal and GSDM-like proteins in bacteria can trigger pyroptosis-like cell death [25,26].

Although studies in evolutionary representatives have suggested the ancient origin and some functional conservations of the GSDM-mediated pyroptosis, the function of GSDM family at the transition from invertebrates to vertebrates is still unclear, remaining the gaps to fully understand the evolution of the GSDM family. Here, we conducted evolutionary analyses of the GSDM family and performed functional analyses of the GSDME homolog in amphioxus, the basal chordate harboring an extraordinarily complex innate immunity. We not only found the highly conserved roles of GSDME-mediated pyroptosis in antibacterial defense in amphioxus, but also shed new light on the feedback regulation of GSDME-mediated inflammatory responses via distinct N termini, alternative splicing and SNPs of GSDME.

## Results

### Evolution of the GSDM family in Metazoans

To shed light on the origin and evolution of GSDM-mediated pyroptosis, we used BLAST programs to search the public genome, expressed sequence tag (EST), and protein databases of the available species. We found that GSDME homologs are widely distributed in multicellular organisms except jellyfish, nematodes, insects, and ascidians (S1 Table). Based on the protein alignment of the GSDM full-length (FL) sequences, we then constructed a maximum-likelihood (ML) tree by taking the GSDM-like molecules from bacteria and fungi as the outgroup (S1 Fig). To better understand the evolution of GSDM family, we also completed the intron phase and gene collinearity analyses of some GSDM representatives among species. In agreement with the previous studies [27,28], the derived tree suggested that GSDMs can be divided into 2 major clades, the GSDMA/B/C/D and GSDME/PJVK clades (S1 Fig). The former is specific to jawed vertebrates, while the latter is distributed widely from cnidaria to mammals (S1 Fig).

**Fig 1:**
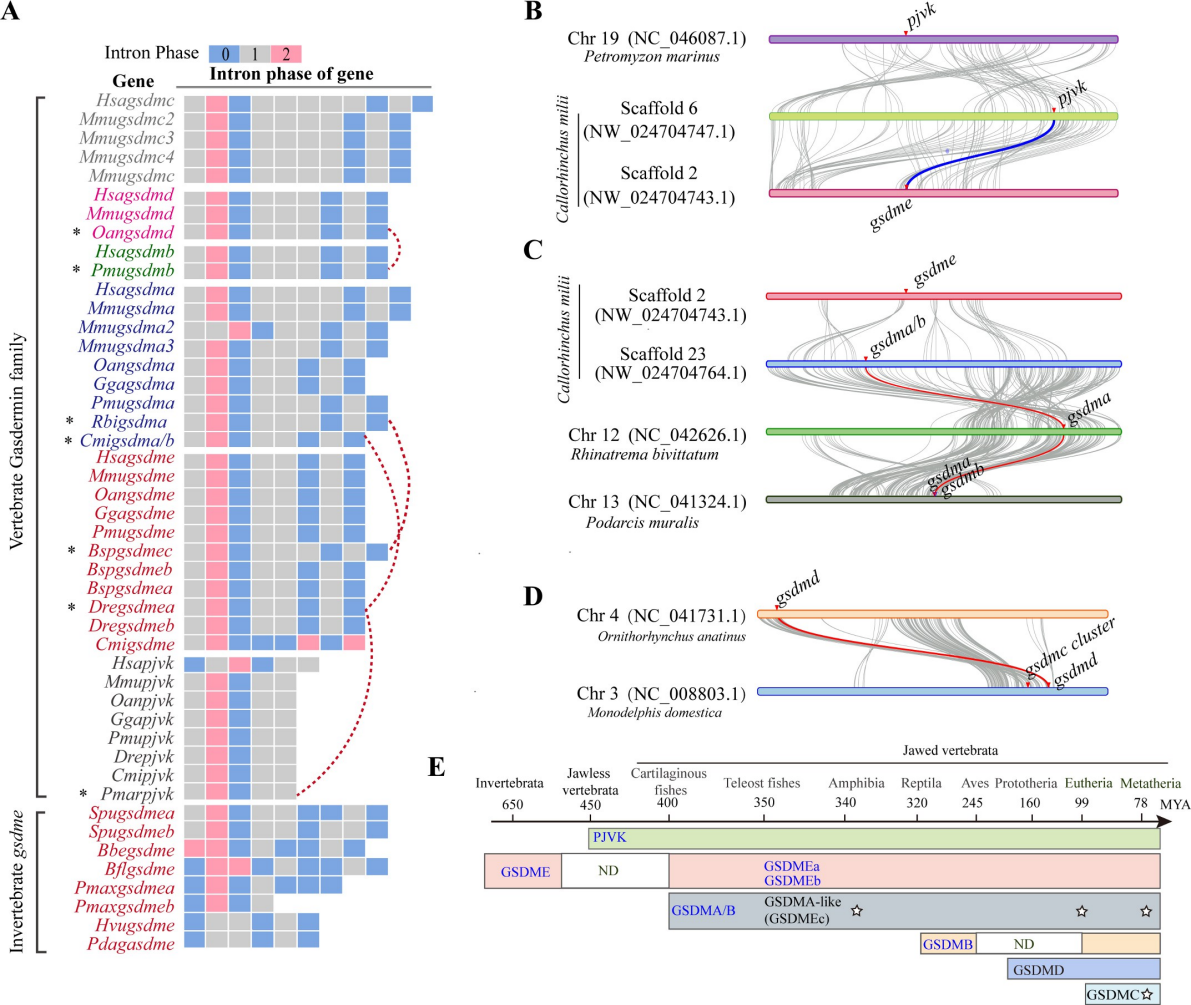
The evolutionary progress of the GSDM representatives. (**A**) The intron phases of some representative *gsdm* genes. Numbers 0, 1, 2 indicate distinct intron phases. The species abbreviations and more details of intron phase were listed in S2 Table. The star and red dotted line mean comparison of intron phase between indicated GSDM genes from 2 species. (**B**) MCScanX is used to analyze the gene linkage and collinearity among the *pjvk* loci of lamprey *Petromyzon marinus* and the *gsdme* and *pjvk* loci of cartilaginous fish *Callorhinchus milii*. The blue line indicates the syntenic relationship between *pjvk* and *gsdme* genes in these 2 species. Gray lines indicate genes with linear relationships. (**C**) MCScanX is used to analyze the gene linkage and collinearity among the loci of *C*. *milii gsdma/b*, *Rhinatrema bivittatum gsdma*, and *Podarcis muralis gsdma*. The red line indicates the syntenic relationship among *Cmigsdma/b*, *Rbigsdma*, and *Pmugsdma*. (**D**) MCScanX is used to analyze the gene linkage and collinearity between the *gsdmd* loci of platypus *Ornithorhynchus anatinus* and another mammal *Monodelphis domestica*. (**E**) Cartoon of the evolution progress of the GSDM family. Colors represent different members of GSDM family. ND indicates that the gene is not identified. The stars indicate the gene expansion of GSDMA and GSDMC in some species.

Intron is a feature that distinguishes eukaryotic gene from prokaryotic gene, and intron phase is an important information to reveal the evolution of genes among species [29,30]. Besides intron phase, using the MCScanX program to analyze gene collinearity is another important data [31,32]. The gene collinearity analyses and intron phase comparison between *D*. *rerio* GSDME and *Petromyzon marinus* (lamprey) *pjvk* indicated that *pjvk* was duplicated from *gsdme* with losing of the last 3 exons, which encode the C-terminal inhibitory domain of GSDME in early vertebrates (Fig 1A and 1B). In addition, *gsdme* may have undergone independent gene expansion or loss during evolution. For example, *gsdme* was lost in jawless vertebrates such as lampreys and hagfish, but duplicated in bony fish and some invertebrates like *P*. *maximus* and *S*. *kowalevskii* (S1 Table). Moreover, the intron phases of invertebrate and cartilaginous fish *gsdmes* are dynamic, indicating the rapid evolution of GSDMEs in these species (Fig 1A and S2 Table).

Unlike PJVK and GSDME, which have clear correlation, the evolutionary footsteps of GSDMA/B/C/D clade are still uncertain. Here, a GSDMA/B homolog was identified from cartilaginous fish like *Callorhinchus milii* and *Rhincodon typus*. This gene is thought to be duplicated from *gsdme*, as it has the same intron phase with that of *D*. *rerio gsdme* (Fig 1A). Using MCScanX program [32], synteny match was identified between *C*. *milii gsdma/b* and *Rhinatrema bivittatum gsdma* (Fig 1C). MCScanX also identified synteny match between *C*. *milii gsdma/b* and *Podarcis muralis gsdmb* (S2A Fig), suggesting that *Cmigsdma/b* is the common ancestor of vertebrate *gsdma* and *gsdmb*. Moreover, in teleost fishes, the intron phase of *gsdmea* and *gsdmeb* was the same as that of other vertebrate *gsdme*, while the intron phase of the recently identified *gsdmec* was more consistent with *gsdma* (Figs 1A and S2B) [33], suggesting that some of the GSDMEc in teleost fishes may function like a GSDMA rather than a GSDME duplicate. Synteny analysis also suggested the correlation between *Anguilla anguilla gsdmec* and *C*. *milii gsdma/b* and *gsdme* (S2C Fig), further suggesting that teleost GSDMEc may be originated from cartilaginous fish GSDMA/B. Besides that, *gsdma* and *gsdmb* have experienced lineage specific evolution, such as the loss of *gsdma* and *gsdmb* in some species. For examples, *gsdma* was lost in some amphibians like *Xenopus laevis*, while *gsdmb* was lost in birds and early mammals. Additionally, the intron phase of reptiles *gsdmb* is different from that of *gsdme* and *gsdma* (Fig 1A and S2 Table), further suggesting the dynamic evolution of GSDMB. Recently, human GSDMB was found to directly lyse intracellular bacteria, and GSDMA can act as a substrate of group A *Streptococcus* (GAS) SpeB and as an effector to trigger pyroptosis [18,19], linking the evolution of *gsdma/b* subgroup to the coevolution of host and species-specific pathogens.

As for mammlian GSDMC/D subgroup, a GSDMD homologue was first found in platypus (*Ornithorhynchus anatinus*), the most primitive mammal. The gene collinearity analysis by MCScanX suggested its orthology with *gsdmd*, indicating that *gsdmd* is more ancient than *gsdmc* during evolution and emerged firstly in early mammals (Fig 1A and 1D). Having the same intron phase further suggested that *gsdmd* may be originated by duplication from *gsdmb* (Fig 1A and S2 Table). Thus, the GSDMA/B homolog identified in cartilaginous fish is the common ancestor of jawed vertebrate GSDMA/B/C/D clade. The gene expansion of GSDMA and GSDMC were also identified in some species. For examples, GSDMA expanded in some amphibians like *Microcaecilia unicolor* and in some mammals like *Monodelphis domestica* and *Mus musculus*. The gene expansion of GSDMC also occurred in *M*. *domestica*, *Equus asinus*, *M*. *musculus*, and *Sus scrofa* (S1 and S2 Tables). Thus, the dynamic evolution of GSDM family should be influenced by the coevolution of host and pathogen. The footstep evolution of GSDM family was summarized, as shown in Fig 1E (Fig 1E).

### BbGSDME triggers pyroptosis and is a substrate of BbCASP 1/2-like and 3-like

**Fig 2:**
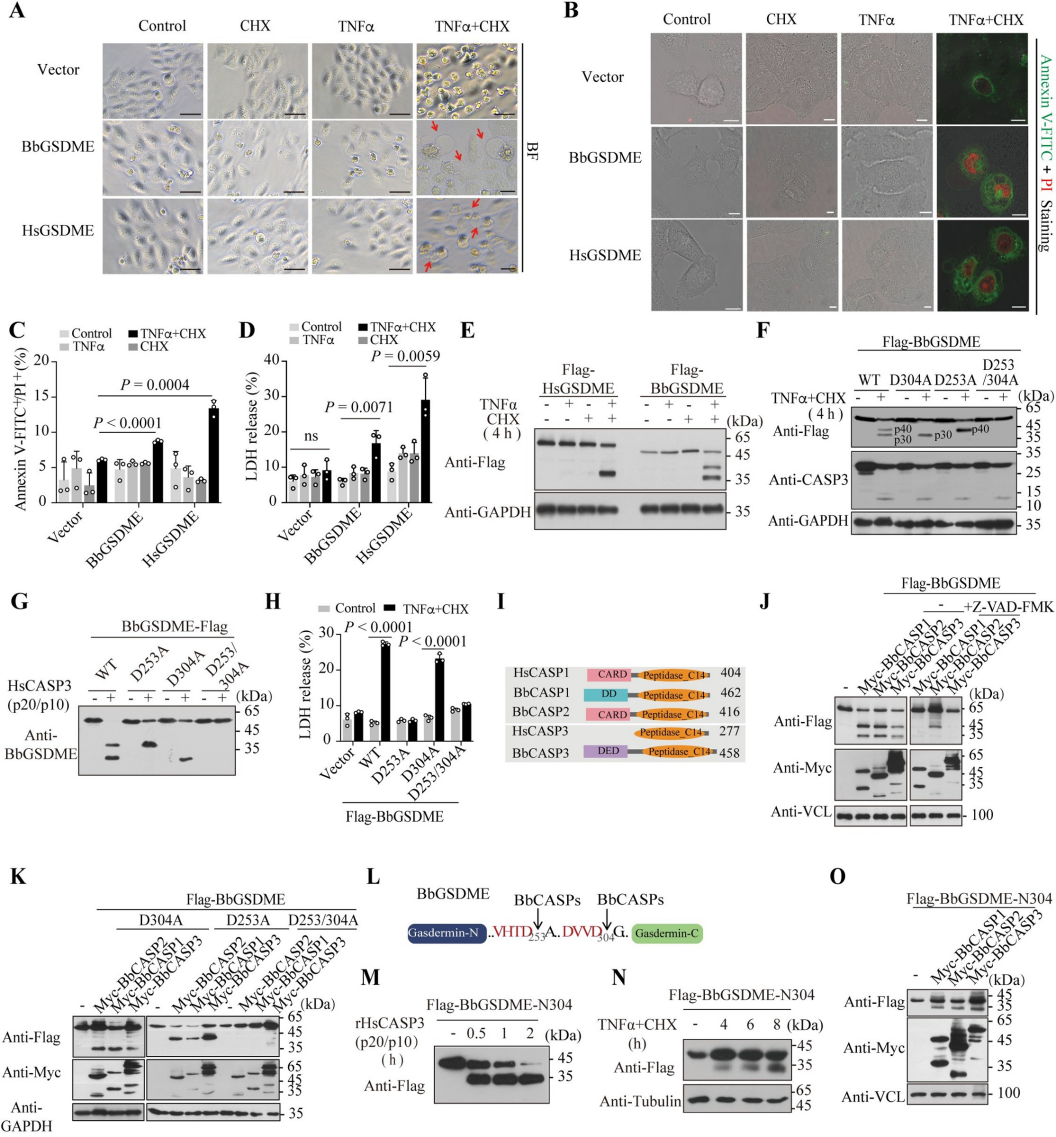
BbGSDME exerted pyroptosis-inducing activities and was a substrate of caspases. (**A**) BbGSDME induced pyroptosis in HeLa^*gsdmd/e DKO*^ upon TNFα plus CHX treatment for 4 h. HeLa^*gsdmd/e DKO*^ cells were transiently transfected with indicated GSDMEs vectors and then stimulated with TNFα (20 ng/ml) alone, CHX (10 μg/ml) alone, or TNFα plus CHX treatment for 4 h. The nature of cell death was assessed by confocal microscopy. Scale bar, 50 μm. (**B**) HeLa^*gsdmd/e DKO*^cells were transiently transfected with indicated GSDM homologs, then treated by TNFα alone, CHX alone, or TNFα plus CHX for 4 h, and then stained with PI and annexin V-FITC. Cell morphology was detected by confocal microscopy. Scale bar, 8 μm. (**C**) Flow cytometry analysis of the PI and annexin V-FITC double staining cells upon indicated transfection and stimulations. Data represent the mean ± SD of 3 independent experiments. *P* values were analyzed with Student’s *t*-test. (**D**) The LDH release mediated by indicated GSDM members in HeLa^*gsdmd/e DKO*^ cells under indicated treatments for 4 h. Triton X-100 treatment was used to achieve 100% LDH release. Data represent the mean ± SD of 3 independent experiments. *P* values were analyzed with Student’s *t*-test. (**E**) The cleaved products of the indicated GSDME homologs in HeLa^*gsdmd/e DKO*^ cells upon indicated stimulations. (**F**) Identification of the CASP cleavage sites in BbGSDME. Constructs of BbGSDME D253A, D304A, or D304/253A mutants were transfected into HeLa^*gsdmd/e DKO*^ cells, which were then treated with TNFα plus CHX. (**G**) Cleavage of BbGSDME and its mutants by active rHsCASP3 in vitro. (**H**) LDH release of HeLa cells, which were transiently transfected with indicated BbGSDME vectors and upon TNFα plus CHX stimulation for 4 h. Data are means ± SD from 3 replicates. Student’s *t*-test. (**I**) Protein architectures of amphioxus CASPs that were used to test the BbGSDME cleavage. (**J**, **K**) Cleavage of BbGSDME (**J**) and BbGSDME mutants (**K**) by BbCASPs at the presence or absence of pan CASP inhibitor Z-VAD-FMK (20 μM) in 293T cells. All the WB and cell morphological images are representatives of at least 3 independent experiments. (**L**) Cartoon diagram of the caspase cleaved sites of BbGSDME. (**M**) In vitro cleavage of BbGSDME-N304 by the active rHsCASP3. (**N**) The cleavage of BbGSDME-N304 upon TNFα plus CHX stimulation in HeLa^*gsdmd/e DKO*^ cells. (**O**) BbGSDME-N304 could be cleaved by BbCASP1-, 2-, and 3-like proteins in 293T cells. Raw data can be found in Supporting information (S1 Raw Images and S1 Values For Plots files). All FCS files are available from the FlowRepository database (Repository ID: FR-FCM-Z642). BF, bright field; CARD, caspase recruitment domain; CHX, cycloheximide; DD, death domain; DED, death effector domain; LDH, lactate dehydrogenase; PI, propidium iodide; TNFα, tumor necrosis factor alpha; WB, western blot.

Above phylogenetic analysis showed that although GSDME is the most ancient GSDM in multiple cellular organisms, it has experienced dynamic evolution in invertebrates and early vertebrates. To fill up the gaps in understanding the functional evolution of GSDM-mediated pyroptosis at the transition from invertebrates to vertebrates, BbGSDME was cloned from the basal chordate amphioxus (*Branchiostoma belcheri*, Bb). Since HeLa cells are sensitive to tumor necrosis factor (TNFα) stimulation and have been used in many GSDM-related studies, BbGSDME was first expressed in *gsdme* and *gsdmd* double-knockout HeLa cells (HeLa^*gsdmd/e DKO*^ cells), and then the cell morphology was observed by microscopy. When cells expressing the FL BbGSDME were treated with TNFα plus cycloheximide (CHX) for 4 h, obvious morphological features of pyroptosis were observed, including swelling with characteristic bubbles formed from the cell membrane as well as loss of membrane integrity (Fig 2A). A significant increase in the number of dead cells stained by both annexin V-FITC and propidium iodide (PI) further suggests that upon TNFα plus CHX treatment, overexpressed BbGSDME can induce pyroptosis in HeLa cells (Figs 2B and 2C and S3A–S3C). The nature of cell death was also confirmed by lactate dehydrogenase (LDH) release (Fig 2D), and the inflammatory response was measured by the detection of interlukin 6 (IL-6) secretion (S3D Fig).

Since TNFα plus CHX treatment can activate caspase 3 (CASP3), to reveal whether BbGSDME can be activated by CASP3 like HsGSDME, western blot (WB) analyses were performed to detect their cleaved products in the HeLa^*gsdmd/e DKO*^ cells upon TNFα plus CHX treatment. Interestingly, as Fig 2E showed, 2 N-termini of approximately 45 kDa and 35 kDa (named p40 and p30, respectively) of BbGSDME were obtained (Fig 2E), indicating that BbGSDME can be proteolytically processed in HeLa cells. Since CASPs cleave specifically at Asp (D) sites of the substrates, 2 potential CASP cleavage sites (Asp at P1) in BbGSDME were predicted according to the sizes of the observed N-fragments, which are D253 and D304. Then, the predicted Asp (D) was mutated to Ala (A), and the cleavage actions were evaluated in HeLa^*gsdmd/e DKO*^ cells upon treatment with TNFα plus CHX. When the D304A mutant form of BbGSDME was overexpressed in HeLa^*gsdmd/e DKO*^ cells, the p40 fragment was barely detectable, while the p30 fragment was significantly increased (Fig 2F). If D253 was replaced with A253, the p40 fragment of BbGSDME significantly increased, while the p30 fragment completely disappeared (Fig 2F). When the D304 and D253 in BbGSDME were both mutated, neither the p30 nor p40 fragment was detected (Fig 2F). To confirm the proteolytically processing of BbGSDME by CASP3, the recombinant BbGSDME and its mutants were prepared and then incubated with the active form of recombinant HsCASP3. As results showed, BbGSDME could be processed by active rHsCASP3 both at the D253 and D304 sites in vitro (Fig 2G), generating the p30 and p40 fragments. Simultaneously, the D304A and D253A double mutant or the D253A single mutant of BbGSDME could not switch TNFα-induced apoptosis to pyroptosis in HeLa^*gsdmd/e DKO*^ cells (Figs 2H and S3E), suggesting that only the p30 fragment could execute pyroptosis in HeLa cells.

In our previous study, we have found gene expansion and dynamic exon shuffling of many immune-related genes in amphioxus [34]. Amphioxus has more than 40 CASPs, including inflammatory CASPs, apoptosis initiator and effector CASPs [35]. To reveal whether amphioxus CASPs may cleave BbGSDME, 2 BbCASP1/2-like and 1 BbCASP3-like homologs were coexpressed with BbGSDME in cells (Fig 2I). The results showed that BbGSDME can be cleaved by all 3 tested BbCASPs to generate the p30 and p40 N-termini (Fig 2J). The cleavage of BbGSDME by BbCASP1-like and BbCASP3-like, but not BbCASP2-like, can be totally inhibited by the pan CASP inhibitor Z-VAD-FMK (Fig 2J). Moreover, when D253 or D304 of BbGSDME was double or single mutated, the expected p30 or p40 fragment cleaved by amphioxus CASPs disappeared (Fig 2K). Thus, these results suggested that the _250_VHTD_253_ and _301_DVVD_304_ are the cleaved sites of BbGSDME, yielding the N253 or N304 termini (Fig 2L). Based on the cleavage sites of BbGSDME, we further found the distribution of VHTD and DVVD motifs in some members of GSDM family, such as DVVD motif in MmuGSDMD and VHTD motif in the N-terminal of MmuGSDMA (S3F and S3G Fig), suggesting that these sites may be potentially recognized by CASPs.

Since the p30 and p40 fragments can be generated by the same CASPs, we further tested whether BbGSDME-N304 can be processed into N253 both in vitro and in HeLa cells expressing amphioxus CASPs. We found that the active rHsCASP3 could process N304 into N253 in vitro (Fig 2M). Consistently, the processing of BbGSDME-N304 into N253 was found upon TNFα plus CHX stimulation in HeLa cells (Fig 2N). In addition, amphioxus CASPs could cleave BbGSDME-N304 to generate the N253 fragment (Fig 2O). Collectively, BbGSDME-N304 is also a substrate of some CASPs, suggesting a new regulation of GSDME mediated pyroptosis.

### BbGSDME-N253 but not N304 can directly mediate pyroptosis

**Fig 3:**
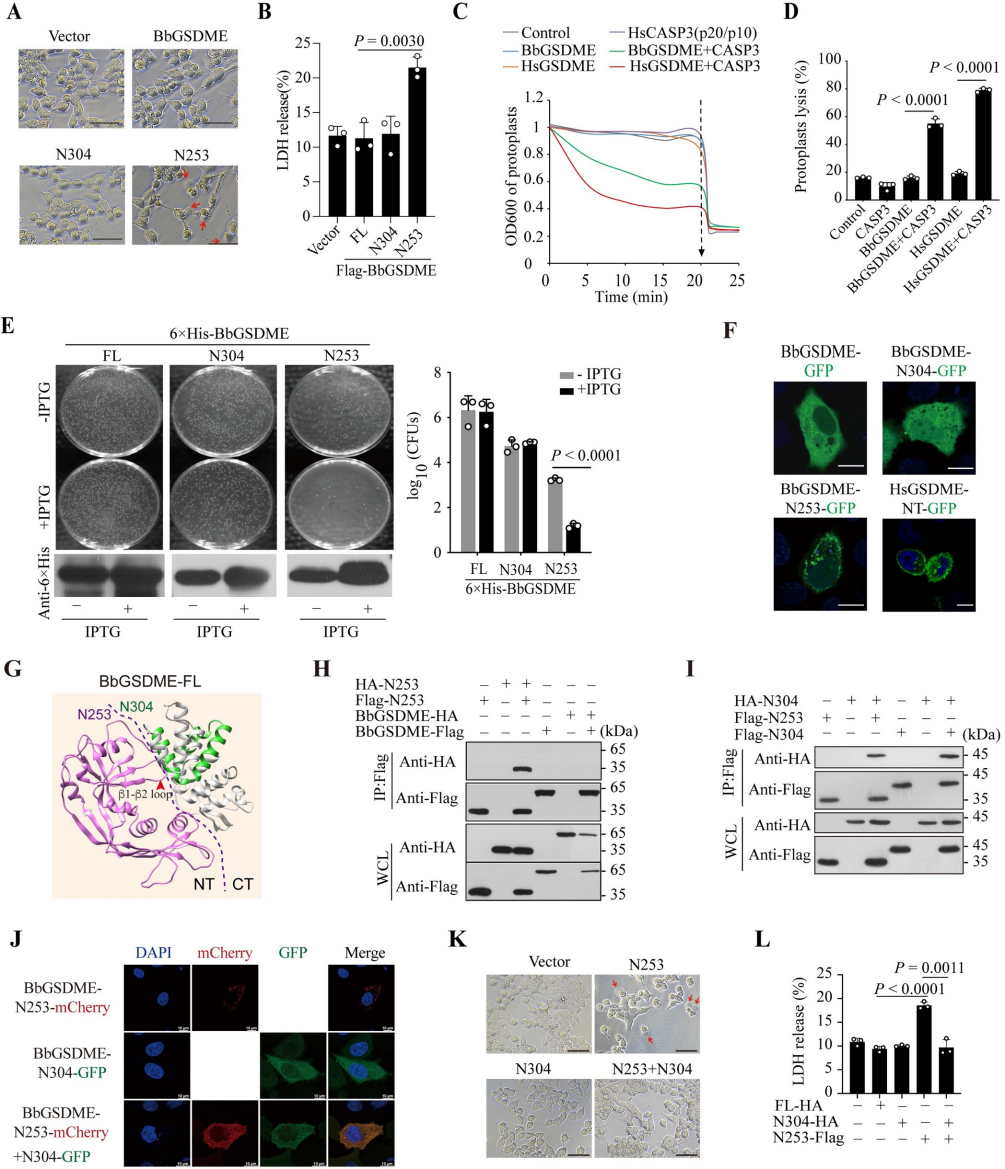
BbGSDME-N253 but not N304 can directly mediate pyroptosis. (**A**) Pyroptotic morphology induced by BbGSDME-N253 and BbGSDME-N304 in 293T cells, which were transiently transfected with indicated GSDMEs vectors. Scale bar, 50 μm. (**B**) Comparison of the LDH release mediated by BbGSDME and its N-termini in 293T cells. Data represent the mean ± SD of 3 independent experiments. *P* values were analyzed with Student’s *t*-test. (**C**) The lytic curve of protoplast. Protoplast leakage by incubating the purified GSDME-FL proteins with protoplasts derived from *B*. *megaterium*. To obtain the active form of GSDME homologs, the FL GSDME proteins purified from HeLa cells were first incubated with the active rHsCASP3 and then added to the derived protoplasts. OD_600_ of *B*. *megaterium* protoplasts was measured every 5 min. (**D**) The ratio of lytic protoplast at 20 min presented in (**C**). Experiments were performed in triplicate. *P* values, Student’s *t*-test. (**E**) Bacteria BL21(DE3) expressing the BbGSDME-FL or its N-termini were cultured on LB plates with or without IPTG induction for 12 h. Expression of the indicated proteins in BL21 were detected by WB using an anti-His mono-antibody. The statistics of the number of colonies on each plate were presented at right. The Y-axis indicated the log _(10)_ (CFUs) on each plate. Data represent the mean ± SD of 3 independent experiments, and *P* values were analyzed with Student’s *t*-test. (**F**) The subcellular localizations of GSDMEs and their N-termini fused with GFP at C-terminus in HeLa cells. Scale bar, 8 μm. (**G**) Overall structure of BbGSDME-FL generated by AlphaFold. The BbGSDME-N and C domains were separated by a dotted line. The purple indicated BbGSDME-N253, and green indicated the 254–304 fragment. (**H**) Co-IP analyses showed that BbGSDME-N253, but not BbGSDME, can self-associate. (**I**) Co-IP analyses showed that BbGSDME-N253 could interact with BbGSDME-N304 and that BbGSDME-N304 could self-associate. Representative images from 3 independent biological experiments were shown. (**J**) The subcellular colocalization of BbGSDME-N253 and N304 fused with GFP or mCherry at C-terminus in HeLa^*gsdmd/e DKO*^ cells. Scale bar, 10 μm. (**K**) 293T cells transfected with indicated vectors were subjected to microscopic observation. Red arrows indicated pyroptotic cells. Scale bar, 50 μm. (**L**) LDH release of 293T cells, which were transfected with indicated constructs. Data represent the mean ± SD of 3 independent experiments. Student’s *t*-test. Raw data can be found in Supporting information (S1 Raw Images and S1 Values For Plots files). BF, bright field; CFU, colony-forming unit; FL, full-length; IPTG, isopropyl β-D-thiogalactoside; LDH, lactate dehydrogenase; WB, western blot; WCL, whole cell lysate.

To validate the functional differences between BbGSDME-N253 and BbGSDME-N304, these 2 fragments were overexpressed in 293T cells and then the cell morphology was observed. The cells expressed BbGSDME-N253 but not BbGSDME-N304 represented apparent pyroptosis morphology (Fig 3A). Concurrently, expression of BbGSDME-N253 but not N304 in 293T cells induced LDH release (Fig 3B). To further test the cytotoxicity of N253, Flag-tagged BbGSDME or HsGSDME were first overexpressed in HeLa^*gsdmd/e DKO*^ cells and purified from the cell lysates. To obtain the GSDME-N termini, the purified GSDME proteins were incubated with the active rHsCASP3. Then, these Flag-tagged GSDME homologs and their CASP3-cleaved products were incubated with protoplasts derived from *B*. *megaterium*. After testing the OD_600_ of the protoplasts, we found that the purified proteins of BbGSDME and HsGSDME FL cannot lyse *B*. *megaterium* protoplasts (Fig 3C and 3D). However, when BbGSDME and HsGSDME were incubated with rHsCASP3 before incubating with protoplasts, lytic effects of the purified proteins on protoplasts could be observed (Fig 3C and 3D).

In addition to protoplast leakage assays, we expressed the BbGSDME and its N-termini in *Escherichia coli* BL21(DE3). Upon isopropyl β-D-thiogalactoside (IPTG) induction, no effect on the growth of BL21(DE3) cells was observed when BbGSDME-N304 and BbGSDME-FL were expressed (Fig 3E). In contrast, in the presence of IPTG, the bacterial clones expressing BbGSDME-N253 proteins were significantly reduced (Fig 3E). Thus, these data highlighted that the BbGSDME-N253 but not the BbGSDME-N304 was capable of pore formation and could inhibit the growth of bacterial cells.

Further analyses of the protein locations by confocal microscopy showed that BbGSDME-N253 could aggregate into dots on the cell membrane, while BbGSDME-FL and BbGSDME-N304 were evenly distributed in the cells (Fig 3F). We then generated the structure model of BbGSDME by AlphaFold, which showed the pore formation domain and the gasdermin-C domain of BbGSDME, similar to mouse GSDMA3 and GSDMD [6,36]. The C-terminus adopted a compact globular fold composed of α-helices and β-strands (Fig 3G). Structural comparison indicated that the β1-β2 loop and the disordered loop in the N-terminus could bind to the region of 254–304 (S4A Fig), suggesting that N304 may present an autoinhibition state. The electrostatic potential map of BbGSDME-N304 in 2 side views suggested that N304 could attract each other through charge interaction of patches I and II, resulting in oligomerization (S4B Fig). Further Co-IP assays confirmed that BbGSDME-N253 and BbGSDME-N304 not only could self-associate, but also interact with each other (Figs 3H, 3I, S4C and S4D). Following confocal imaging showed that when N253 was coexpressed with N304, its cell membrane targeting was disrupted by colocalization with N304 (Fig 3J), suggesting that N304 may serve as a negative regulator of BbGSDME-mediated pyroptosis. To address this issue, we coexpressed N253 with N304 in 293T cells and found that the N253-mediated pyroptosis and the release of LDH could be inhibited (Fig 3K and 3L).

Besides cleavage by distinct CASPs to generate N-termini with different functions, fragmented BbGSDME can be produced by alternative splicing. During cDNA cloning, 6 *Bbgsdme* alternative splicing isoforms were found from amphioxus gill slits, muscle, and skin. *Bbgsdme-S1* lacking the C-terminal inhibitory domain due to early termination of protein translation was obtained from amphioxus gill slits, while *Bbgsdme-S2* to *Bbgsdme-S6* containing partial N-terminal pore-forming domain and/or partial C-terminal inhibitory domain were isolated from amphioxus muscle and skin (S4E and S4F and S5 Figs). LDH release assay and cell morphological observation indicated that BbGSDME-S1 but not BbGSDME-S2 could induce pyroptosis in 293T cells (S4G and S4H Fig). Thus, *Bbgsdme* generated by alternative splicing may increase the regulatory complexity to control the excessive inflammatory response mediated by GSDME in amphioxus.

### BbGSDME may participate in muscle necrosis upon bacterial infection and is transcriptionally regulated by BbIRF1/8

**Fig 4:**
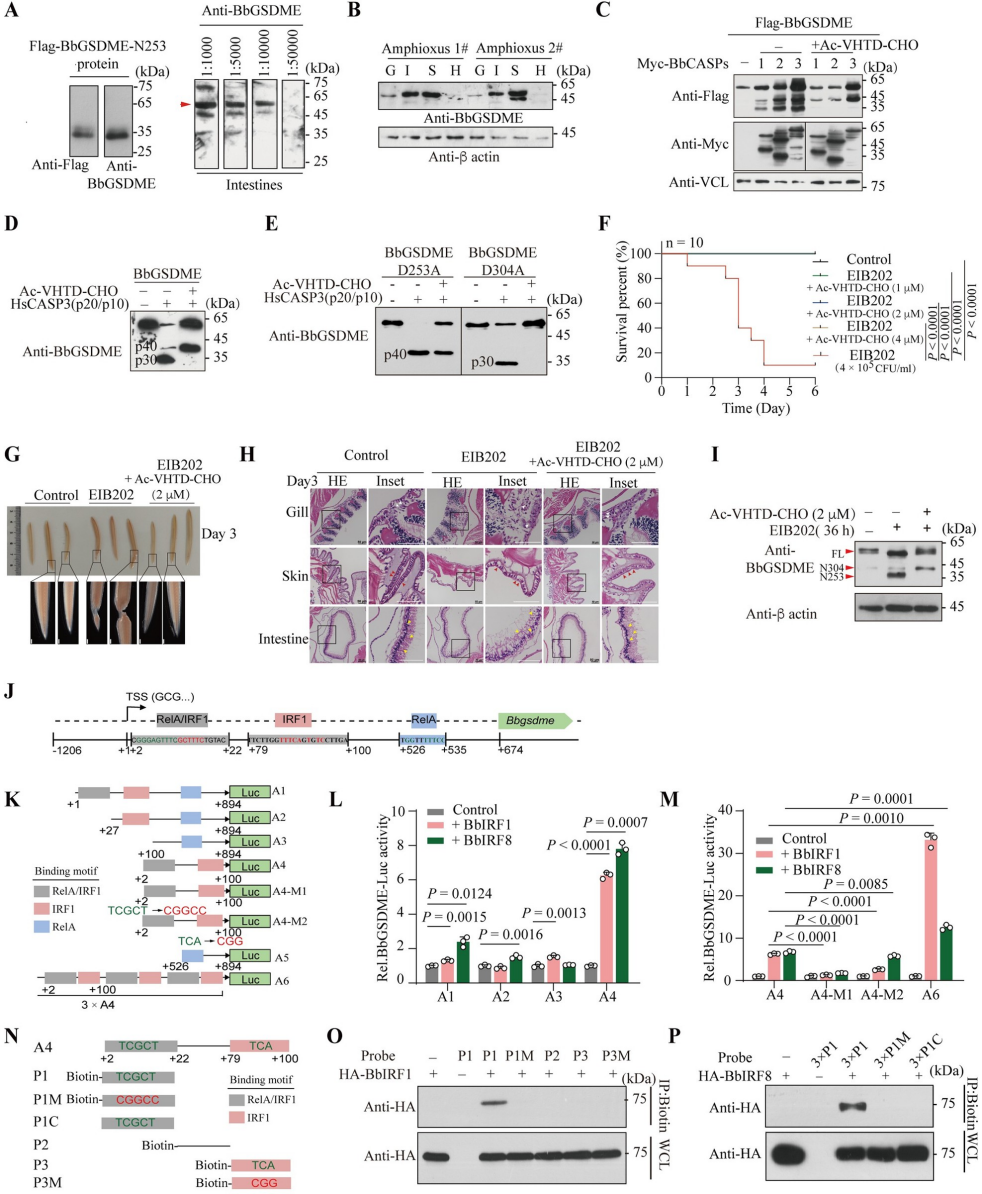
BbGSDME may participate in bacterial resistance and muscle necrosis in amphioxus. (**A**) WB assays to detect the binding specificity of the anti-BbGSDME serum to the purified Flag-tagged BbGSDME-N253 (left) or the total protein extracted from amphioxus intestines (right). (**B**) WB assays to detect the abundance of BbGSDME in amphioxus tissues. (**C**, **D**) Ac-VHTD-CHO inhibited the cleavage of BbGSDME by BbCASP1-, 2-, and 3-like in 293T cells (**C**) or by the active form of rHsCASP3 in vitro (**D**). (**E**) Ac-VHTD-CHO inhibited the cleavage of BbGSDME-D304A by the active form of rHsCASP3 in vitro. (**F**) Surviving curve for amphioxus adults infected with EIB202 in the presence or absence of Ac-VHTD-CHO. *P* values were calculated by the log-rank (Mantel–Cox) test method. (**G**) The morphology of amphioxus individuals postinfection by EIB202 in day 3 at the presence or absence of Ac-VHTD-CHO. White scale bar is 500 μm. (**H**) Representative HE staining images of the gill slits, skin, and intestines of healthy and EIB202-infected amphioxus. Images were obtained from EIB202-infected or EIB202 plus Ac-VHTD-CHO-treated amphioxus on day 3 postinfection. Red arrows indicate the ciliated single layer columnar epithelial cells of skin. White arrows indicate the arranged columnar epithelial cells of gill filaments. Yellow arrows indicate the arrangement of columnar cells in intestines. Scale bar, 50 μm. (**I**) The cleavage of BbGSDME in EIB202-infected amphioxus intestines on 36 h postinfection. (**J**) The predicted binding motifs of IRF1 and RelA at *Bbgsdme* promoter region. (**K**) Schematic diagram of the reporter constructs. The A4-M1 and A4-M2 indicated constructs with mutations at the 5′-TCGCT-3′ element or the 5′-TCA-3′ element. The A6 construct contains 3 repeats of the segment presented in A4. (**L**) The dual-luciferase reporter assays indicated that BbIRF1 and BbIRF8 could induce the expression of reporter in 293T cells. (**M**) The dual-luciferase reporter assay showed that mutations of the RelA/IRF1 binding sites could abolish the transcription of reporter gene activated by BbIRF1/8. However, triplicating the RelA/IRF1 binding site can significantly increase the transcription of reporter at the presence of BbIRF1/8. Data represent the mean ± SD of 3 independent experiments. *P* values were analyzed with Student’s *t*-test. (**N**) Schematic diagram of the probes for DNA pull-down assays. Biotin-labeled probes (P1, P2, and P3) were derived from A4 construct. P1C indicated the competitive probe, which was unlabeled. P1M and P3M indicated biotin-labeled probes with mutations at the 5′-TCGCT-3′ element or the 5′-TCA-3′ element. (**O**, **P**) DNA pull-down assay to detect the direct binding of BbIRF1 (**O**) and BbIRF8 (**P**) proteins to the indicated probes. The streptavidin beads bound DNA-protein complex were detected by WB. Data were representative of 3 independent experiments. Raw data can be found in Supporting information (S1 Raw Images and S1 Values For Plots files). CFU, colony-forming unit; G, gill; H, hepatic caecum; HE, hematoxylin–eosin; I, intestines; IRF1, interferon regulatory factor 1; IRF8, interferon regulatory factor 8; S, skin; TSS, transcription start site; WB, western blot; WCL, whole cell lysate.

To understand the biological roles of the CASP-GSDM axis at the transition from invertebrates to vertebrates, we first detected the temporal–spatial expression of *Bbgsdme* using whole-mount in situ hybridization and real-time PCR. Although no specific distribution during amphioxus embryogenesis was observed for *Bbgsdme* (S6A Fig), it was found to be abundant in adult gill, intestines, and skin, the first line of immune defense of amphioxus (S6B Fig). When we collect amphioxus using fishing tools in the field, it often leads to epidermal damage and bacterial infection. Once this happens, the infected amphioxus will soon have muscle necrosis and dissolution (S6C Fig). We speculated that this phenomenon may be related to the activation of BbGSDME. To connect bacterial infection and GSDME activation in the amphioxus, we generated a polyclonal antibody using the His-tagged BbGSDME protein expressed in BL21 as an antigen (S6D Fig). Then, the purified Flag-tagged BbGSDME protein and the total protein extracted from amphioxus intestines were used to detect the titer and specificity of this anti-BbGSDME antiserum (Figs 4A and S6E). As results showed, 1:10,000 was an optimal diluted concentration to specifically detect the BbGSDME proteins from amphioxus intestines or 293T cells (Figs 4A and S6E). WB assays also confirmed that this antiserum did not cross react with other GSDM proteins (S6F Fig). Using this antiserum, we confirmed the abundance of BbGSDME protein in amphioxus gill slits, intestines, and skin (Fig 4B). Then, bacteria *Edwardsiella tarda* (strain EIB202) or EIB202 together with Z-VAD-FMK were added to the filtered seawater to culture healthy amphioxus. EIB202 is an intracellular gram-negative bacterial pathogen that can infect marine and freshwater fishes, resulting in fish diseases in aquaculture industries worldwide and accounting for severe financial losses [37]. The results showed that 2 μM Z-VAD-FMK could significantly slow the mortality of amphioxus infected by EIB202 (S6G Fig). Microscopic observation further showed that the severe skin ulceration and muscle necrosis of the infected individuals were indeed greatly alleviated by Z-VAD-FMK (S6H Fig).

Z-VAD-FMK is a pan-caspase inhibitor that targets most caspases. There are thousands of potential substrates of caspase family, which play critical roles in maintaining homeostasis, may be affected by Z-VAD-FMK [3]. To avoid such side effects, we then generated the peptide inhibitor Ac-VHTD-CHO, which is derived from the _250_VHTD_253_ cleavage site. We first confirmed that Ac-VHTD-CHO can inhibit the cleavage of BbGSDME at D253 by active rHsCASP3 in vitro and by amphioxus CASPs in 293T cells (Fig 4C and 4D). Moreover, we purified the proteins of BbGSDME-D304A and BbGSDME-D253A mutants from 293T cells and then performed in vitro cleavage using active rHsCASP3. Results showed that the generation of N253 from the BbGSDME-D304A mutant was completely inhibited by the Ac-VHTD-CHO inhibitor, while the generation of N304 from the BbGSDME-D253A mutant could not be abolished (Fig 4E). Consistently, when the DMPD cleavage site in human GSDME was replaced as DVVD or VHTD, the cleavage at the VHTD but not DVVD site was inhibited by the Ac-VHTD-CHO inhibitor in vitro (S6I Fig), suggesting that Ac-VHTD-CHO should have high substrate specificity. To further reveal the side effect of Ac-VHTD-CHO inhibitor by targeting the other proteins that contain the VHTD motif, we then searched the DVVD and VHTD motifs in amphioxus (*Branchiostoma floridae*) proteins database. As the result shows, there are 59 proteins containing the VHTD motif in amphioxus (S6J Fig and S3 Table). Previous study has suggested that the cleavage efficiency of CASPs depends not only on the cleavage motif, but also on the secondary structure and the types of P’1 residue (A/G/S residue) of the substrate [38]. Further analysis of the P’1 residue in proteins containing VHTD motif showed that the VHTDA/G/S motif existed in only 15 proteins, suggesting a small set of substrates potentially cleaved by amphioxus CASPs at the VHTD motif. Among these 15 proteins, no one is related to inflammatory response based on the existing literatures (S6J Fig and S3 Table). Thus, we suggested that the Ac-VHTD-CHO inhibitor can specifically inhibit the generation of BbGSDME-N253 fragment and has little side effect on the other physiological process in amphioxus.

Similar to the observation in Z-VAD-FMK treatment, pretreatment with Ac-VHTD-CHO significantly protects adult amphioxus from bacterial infection at the concentration of 1 μM (Fig 4F). Moreover, the Ac-VHTD-CHO could attenuate the muscle lysis and skin ulceration of the infected individuals (Fig 4G). Hematoxylin–eosin (HE) staining further confirmed that EIB202 infection led to the destruction of tissue integrity of amphioxus pharyngeal gill slits, skin, and intestines (Fig 4H). Upon 2 μM Ac-VHTD-CHO treatment, the vacuolation and necrosis of the columnar epithelial cells arrayed on the surface of gill slits could be significantly alleviated (Fig 4H). Similar effect by Ac-VHTD-CHO on the shrinking and vacuolation of amphioxus skin columnar epithelial cells was also observed (Fig 4H). Additionally, treatment with Ac-VHTD-CHO greatly remained the integrity of intestinal epithelial cells and the arrangement of intestinal monolayer columnar cells (Fig 4H). Moreover, using the BbGSDME polyclonal antibody, we confirmed that the cleavage of BbGSDME at D253 was inhibited by Ac-VHTD-CHO in amphioxus intestines upon challenge with EIB202 (Fig 4I). Thus, the Ac-VHTD-CHO peptide may be a useful compound to resist inflammatory reactions mediated by BbGSDME activation in amphioxus.

Since BbGSDME is abundant in immune-related tissues and is related to bacterial infections, to further reveal the transcriptional regulation of *Bbgsdme*, we then performed bioinformatics analysis using JASPAR (http://jaspar.genereg.net/) to identify the region from −1,206 to +674 on the *Bbgsdme* promoter that contains interferon regulatory factor 1 (IRF1) or RelA binding motifs (Fig 4J). Based on these analyses, reporter constructs containing the region from −1,206 and +674 and the truncated mutants with deletions of 1 or 2 conserved motifs were generated (Fig 4K). Next, these reporter constructs were transfected with HA-tagged BbIRF1, BbIRF8 and Bbp65 expression plasmids into 293T cells. BbIRF1, BbIRF8, and Bbp65 have been found to be transcriptional activators that bind to the IFN-stimulated response element (ISRE) and kappa B (κB) motifs, respectively, in our previous studies [39,40]. Reporter assays showed that BbIRF1 or BbIRF8 could slightly activate the transcription of reporter that contains the *Bbgsdme* promoter by transfecting the A1 construct into 293T cells (Fig 4L). However, when the A4 reporter construct, which contains a sequence between +2 and +100 was transfected with BbIRF1 and BbIRF8, the expression of the reporter gene was significantly up-regulated (Fig 4L). Moreover, when the region from +2 to +100 was 3-fold assembled, the expression of the reporter gene was markedly up-regulated in the presence of BbIRF1 or BbIRF8 (Fig 4M). However, mutations of the 5′-TCGCT-3′ or the 5′-TCA-3′ motifs located from +2 to +100 completely abolished the expression of the reporter gene (Fig 4M). In addition to BbIRF1/8, reporter assays showed that the expression of *Bbgsdme* may be also slightly regulated by Bbp65 (S6K Fig). To further confirm whether BbIRF1/8 could directly bind to the regulatory element within the *Bbgsdme* promoter, we performed DNA pull-down assays. As results showed, BbIRF1 and IRF8 could directly bind to the IRF1/8 binding motif on the promoter of *Bbgsdme* (Fig 4N–P). However, in consistent with the reporter assay, DNA pull-down assays showed that the binding activity of BbIRF8 was lower than BbIRF1, as BbIRF8 could only bind to the probe with triple motifs (Fig 4O and 4P). Thus, *Bbgsdme* can be transcriptionally regulated by BbIRF1/8, which further suggests its immune relevance.

### The residues that are involved in lipids binding and oligomerization of GSDM family are highly conserved during evolution

**Fig 5:**
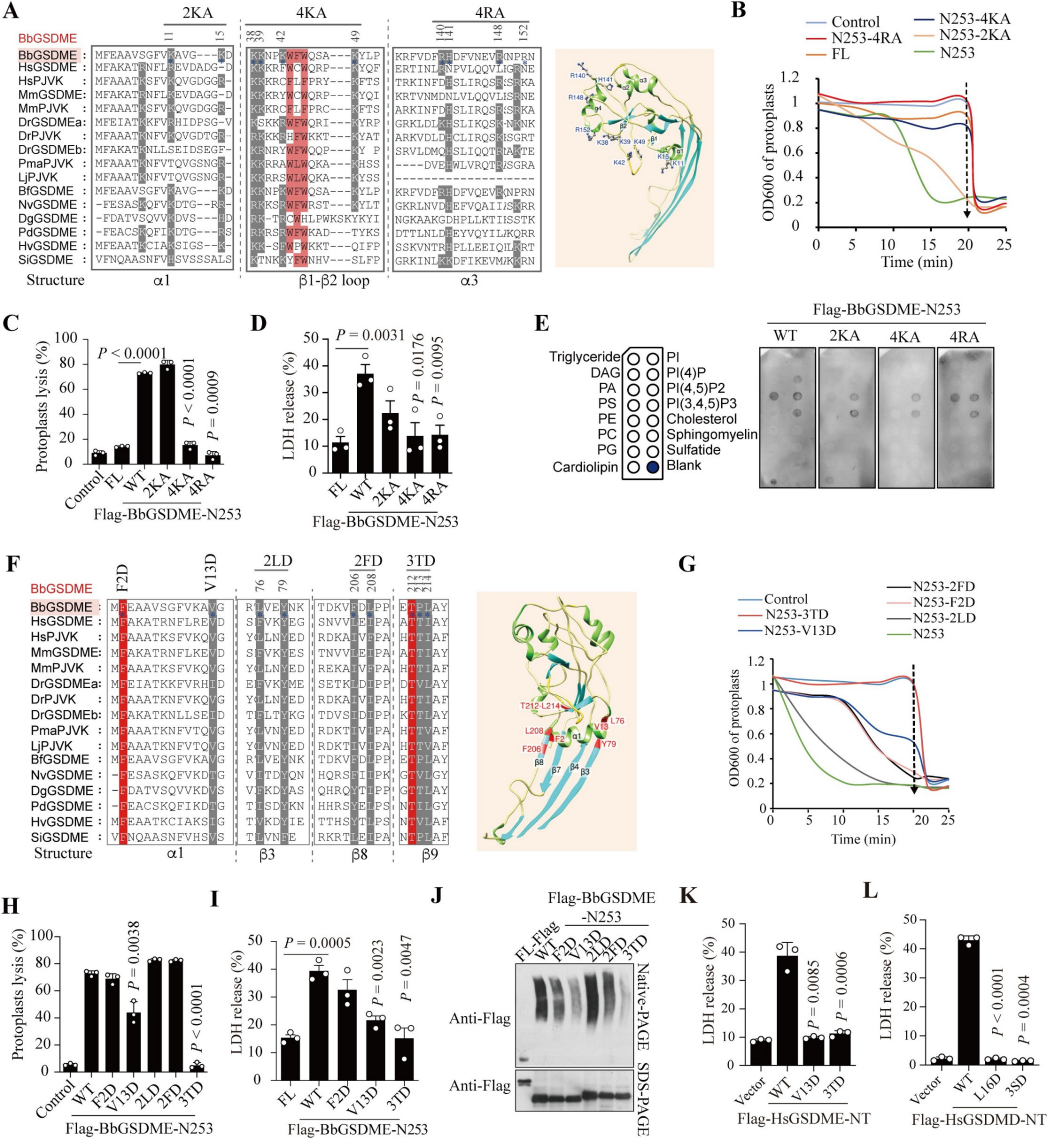
The evolutionarily conserved sites that are involved in lipid binding and oligomerization of BbGSDME. (**A**) Multiple sequence alignment of the GSDME and PJVK proteins showed the positively charged residues (gray) and the aromatic sites (pink). Blue stars highlight the mutated amino acids in BbGSDME. (**B**, **C**) The percentage of protoplast lysis mediated by purified BbGSDME, BbGSDME-N253 and its mutants. *n =* 3, Student’s *t*-test. (**D**) The release of LDH from 293T cells expressing indicated vectors was determined. *n* = 3, Student’s *t*-test. (**E**) Lipids strip binding assays showed that the 4KA mutant can reduce the binding of BbGSDME-N253 to PI(4)P, cardiolipin, and PA, while the 4RA mutant can reduce the binding to PI(4)P and cardiolipin. (**F**) Multiple sequence alignment of GSDME and PJVK proteins showed the highly conserved residues (red) and the ambiguous sites (gray) that may be responsible for oligomerization. Blue stars highlight the mutated residues in BbGSDME-N253. (**G**, **H**) Protoplast lysis mediated by purified BbGSDME-N253 and its indicated mutants. *n* = 3, Student’s *t*-test. (**I**) The LDH release mediated by BbGSDME-N253 and its mutants was tested using 293T cells. (**J**) Electrophoretic analysis of BbGSDME-FL, BbGSDME-N253, and its mutants by 6% native PAGE showed that mutants of 3TD and V13D could reduce the self-association of BbGSDME-N253. Representative images from 3 independent biological experiments were shown. (**K**) The LDH release mediated by HsGSDME-NT and its mutants was tested using 293T cells. (**L**) The LDH release mediated by HsGSDMD-NT and its mutants was tested in 293T cells. Raw data can be found in Supporting information (S1 Raw Images and S1 Values For Plots files). DAG, diacylglycerol; LDH, lactate dehydrogenase; PA, phosphatidic acid; PC, phosphatidylcholine; PG, phosphatidylglycerol; PI, phosphatidylinositol; PI(4)P, phosphatidylinositol(4)-phosphate; PI(4,5)P2, phosphatidylinositol(4,5)-bisphosphate; PI(3,4,5)P3, phosphatidylinositol(3,4,5)-trisphosphate; PS, phosphatidylserine; PE, phosphatidylethanolamine; PJVK, Pejvakin; Sulfatide, 3-sulfogalactosylceramide.

To further reveal the functional evolution of GSDME, evolutionarily conserved, positively charged residues among the GSDME homologs from 12 distinct species were identified using MAFFT. Moreover, the predicted 3D structure of BbGSDME was simulated based on the HsGSDMD (PDB: 6vfe.1) models [41,42] (S7A Fig). Alignment and structure simulation showed that 3 lipid-binding sites with positively charged residues in mammalian GSDMD [6,7,36], including α1 (K7, K10, K14), α3 (R138, K146, R152, R154), and β1-β2 loops (R43, K44), are all conserved in GSDME and PJVK (Fig 5A). Due to their potential importance, we engineered mutant forms of BbGSDME-N253 and used *B*. *megaterium* protoplasts to test their lytic activities. The results showed that when K38, K39, K42, and K49 in the β1-β2 loop were all mutated to Ala (named 4KA), the protoplasts could not be lysed (Fig 5B and 5C). Similar results were obtained when R140, H141, R148, and R152 in α3 were all mutated to Ala (named 4RA) (Fig 5B and 5C). However, the 2KA mutant of BbGSDME-N253 (K11 and K15 in α1 were mutated as Ala) can lyse protoplasts similar to BbGSDME-N253 (Fig 5B and 5C). Concurrently, we observed that 4KA and 4RA reduced LDH release in 293T cells (Fig 5D). Furthermore, protein-lipid strip assays showed that the 4KA mutant disrupted the binding ability to cardiolipin, PI(4)P, and PA, while the 4RA mutant reduced the binding to PI(4)P and cardiolipin (Fig 5E). These results implied that the positively charged residues in the α1 helix, α3 helix, and β1-β2 loop played essential roles in lipid binding of BbGSDME. Since cardiolipin is one of the major bacterial lipids, the results further suggested that the lytic effect of GSDME-N on protoplasts may be dependent on its binding to cardiolipin.

Previous studies have demonstrated that the β-sheets of GSDM proteins play a role in forming oligomers [36,43]. Thus, several conserved amino acids in the α1 helix and β3-, β8-, and β9-sheets of BbGSDME were mutated, and protoplasts of *B*. *megaterium* were used to test their lytic activity (Fig 5F). The results found that when T212-214 in the β9 sheet was mutated to Asp (named 3TD) or V13 in the α1 helix was mutated to Asp (named V13D), the percentages of lytic protoplasts were significantly reduced (Fig 5G and 5H). Consistently, V13D and 3TD effectively blocked LDH release, while F2D did not (Fig 5I). However, unlike human GSDME, whose F2 in the α1 helix may play a critical role in forming oligomers [44], the F2D mutant did not affect the lytic activity of BbGSDME-N253 (Fig 5G and 5H). Similarily, the L76D and Y79D double mutant (named 2LD) or the F206D and L208D double mutant (named 2FD) did not reduce the percentage of lytic protoplasts, thus uncovering a different mechanism by which invertebrate GSDME might be regulated (Fig 5G and 5H).

Since V13 and T212-214 are located at the interface of the BbGSDME pore subunit, we next used native PAGE to examine whether V13D and 3TD mutants inhibit the oligomerization of BbGSDME-N253. The results clearly showed that the oligomer-forming activity of BbGSDME-N253 was reduced when V13 and T212-214 were mutated to Asp (Fig 5J). Due to the high conservation of V13 and T212-214 among distinct GSDM proteins, we further constructed mutants of HsGSDME-NT (V13 and T215-217). As results showed, the pyroptotic phenotype and the release of LDH were significantly suppressed when the V13 and T215-217 in HsGSDME were mutated into aspartates (Figs 5K and S7B). Consistently, when the corresponding residues, L16 and S212-214 in HsGSDMD were mutated into aspartates, the GSDMD-mediated pyroptosis was abolished in 293T cells expressing the indicated mutants (Figs 5L, S7C and S7D). The structure model of GSDMD pore also shows that L16 and S212-T213 are located at the interface of the GSDMD pore subunit (S7E Fig), uncovering the amino acid basis of oligomerization among different GSDMs. Recently, the phosphorylation of GSDMD-NT at Thr213 was identified and its phosphoric mutation can impair the GSDMD-mediated pyroptosis [45]. Using NetPhos online prediction tool, we found that the conserved T215 in GSDME may also be a potential phosphorylation site, suggesting that phosphorylation at T215 may be critical for the function of GSDME during evolution.

To obtain more insight into the key residues of GSDME in physiological state, we analyzed the single nucleotide variants using the public database ClinVar. Based on the sequence alignment of PJVK and GSDME, we then focused on 8 mutations with clinically relevance and highly evolutionary conservation (S7F Fig and S1 Appendix). Functional comparison showed that 2 mutants of HsGSDME, the K120Q and P212L, attenuated the LDH release and disrupted morphology of pyroptosis (S7G and S7H Fig). Using ubibrowser website, the K120 in HsGSDME was predicted to be a potential site for ubiquitination, suggesting that ubiquitination modification at this site may be important for the pyroptotic function of GSDME.

As above results showed, some evolutionarily conserved amino acids are crucial for the functions of GSDME. Druing the cloning of *Bbgsdme*, single nucleotide polymorphisms (SNPs) were also observed for BbGSDME in addition to having alternative splice isoforms. After analyzing 8 FL *Bbgsdme* sequences, a total of 3.61% SNP frequency of *Bbgsdme* among individuals was obtained (S7I Fig). Approximately 15% of the identified SNP sites were nonsynonymous and may result in the functional alteration of BbGSDME (S7J Fig). For example, A48>P in the β1-β2 loop, M63>K in the loop, and N145>K in the α3 helix may alter the lipid binding or oligomerization of BbGSDME (S7K and S7L Fig). SNPs of GSDMB were first realized to associate with asthma [46,47]. Recently, SNPs of GSDMD were found to alter GSDMD function from conserving normal pyroptotic function to inhibiting caspase cleavage to disrupting oligomerization and pore formation [48]. Thus, SNPs of *Bbgsdme* may alter its function to avoid excessive inflammatory response. Comparative analysis based on the evolutionary aspect may be an important way to identify disease-related SNPs and to understand the regulation of GSDM-mediated pyroptosis.

## Discussion

### Targeting GSDM execute pyroptosis should be an important means in controlling microbial infection during evolution

Pyroptosis, a new type of programmed cell death mediated by GSDM, is characterized by the swelling and rupture of cells, release of cellular contents, and a strong inflammatory response [12]. GSDM-mediated pyroptosis has been shown to be critical for controlling microbial infection and tumor growth in mammals [12]. Recently, a coral GSDME homolog was reported to be cleaved by coral CASP3 to induce pyroptosis [24]. Coral can be protected from bacterial infection when coral GSDME cleavage was inhibited by a CASP3 inhibitor [24]. By establishing a zebrafish crispant in vivo analysis model, Yang’s group revealed that the zebrafish caspy2-GSDMEb axis contributes to lethal LPS-induced septic shock and NETosis for bacterial clearance in vivo [21,49]. Similar to its counterparts, amphioxus BbGSDME is cleaved by CASPs to release its BbGSDME-N253, which can form oligomers and bind to membrane lipids in a similar fashion as mammalian GSDMD-NT. Moreover, cleavage of BbGSDME and tissue damage induced by bacterial infection can be alleviated by the pan CASP inhibitor Z-VAD-FMK and the specific peptide Ac-VHTD-CHO in amphioxus. Since GSDMD emerged for the first time in primitive mammals, studies from coral, amphioxus, and zebrafish GSDME indicated that GSDME may concurrently function as mammalian GSDMD in bony fish and other aquatic invertebrates [24,49].

Besides cleavage by CASPs, some GSDMs are the substrates of pathogens proteases. For instance, GSDMA can trigger pyroptosis after cleavage by SpeB, a protease of *streptococcus pyogenes*, which helps the host to recognize and control virulence of bacterial pathogens [19]. Porcine GSDMD can be cleaved by Seneca Valley Virus 3C protease and Enterovirus 71 3C protease at Q193 and Q277 [50,51]. Cleavage at Q277 can trigger pyroptosis, which facilitates viral replication and production [50]. These studies highlighted the potential for the treatment of bacterial infection by inhibitors of protease. Recently, inhibition of human GSDMD cleavage by Ac-FLTD-CMK has been shown to suppress pyroptosis downstream of both canonical and noncanonical inflammasomes and reduce IL-1β release following activation of the NLRP3 inflammasome in macrophages [52]. Besides in mammals, the Ac-FEID-CMK inhibitor derived from the processing site of zebrafish GSDMEb can suppress the noncanonical inflammasome pathway in vivo septic shock model in zebrafish [49]. Here, we showed that the Ac-VHTD-CHO peptide could reduce the mortality of infected lancelets by reducing tissue damage. Since GSDME homologs are widely distributed in aquatic animals, targeting GSDMEs or their executed proteases like CASPs may provide new targets for the treatment of bacterial infection in economically important animals in the process of aquatic culture. It is worth noting that high concentration of Z-VAD-FMK (4 μM in this study) did not have a better effect on alleviating amphioxus death upon bacterial infection. Sine CASP cleavage sites usually are member specific and even lineage specific [23,33], generating the peptide inhibitor based on analyzing the cleavage sites is more efficient and may avoid other side effects.

### Activation and transcriptional regulation of GSDM proteins during evolution

Pathogen-induced plasma membrane rupture is important for the arm race between host and pathogen. Pathogens can disseminate through lytic cell death, while the hosts utilize this mechanism to accelerate immune responses. For example, *Gsdmd* deficiency promoted host defense against *C*. *albican* infection as it prevented macrophages from lysis cell death and attenuated the escape of pathogen [53]. However, inhibiting the cleavage of turbot GSDMEa significantly decreased the survival of turbot upon *V*. *harveyi* infection [22]. Here, we observed that disruption of the GSDME cleavage in amphioxus protected amphioxus from bacteria-induced tissue necrosis. Since protein-lipid strip assays showed that BbGSDME could weakly bind to cardiolipin, which is an essential component of gram-negative bacterial membranes, to determine if BbGSDME selectively targets bacteria, we assessed the viability of EIB202 exposed to BbGSDME, HsGSDMB, and their N-terminus in vitro. Coincubation of HsGSDMB-NT, but not BbGSDME-N253 or their FL proteins, could lyse EIB202 (S8A Fig). Thus, the protection of amphioxus from EIB202 infection by Ac-VHTD-CHO may be due to the inhibition of BbGSDME-N253-mediated inflammation, but not directly bacteria targeting. We have also immersed adult amphioxus with lipopolysaccharide (LPS) from *E*. *coli* O111:B4 for 4 h or 8 h, but no significant cleavage of BbGSDME-N253 was observed (S8B and S8C Fig). Since EIB202 is an intracellular bacterium (S8D Fig), we thought that the Caspase-GSDME signaling should be activated by the infection of intracellular bacteria or the recognition of intracellular LPS. Although there are up to 92 NLR genes and 45 CASPs in amphioxus due to the expansion of innate immune-related genes [34], how intracellular LPS is recognized is still unclear at present. After sequence analyses, we believed that an inflammasome-like complex may be formed by some BbNLRs, BbCaspase1/2, BbASCs based on the death effector domain (DED) interaction in amphioxus (S8E Fig). However, such hypothesis needs further elucidation, because of the lack of amphioxus cell lines and antibodies at present.

Mammalian GSDM members have variable tissue and cell distributions. For example, mammalian GSDME is variably expressed in the brain, endometrium, placenta, and intestine, while PJVK is highly expressed in the testis and broadly expressed in hair cells of the inner ear and other cells of the auditory system (S8F Fig) [12]. Recently, transcription of *gsdmd* was shown to be up-regulated in DCs in response to microbial c-di-GMP and *Salmonella typhimurium* infection upon activation of IRF1 [54]. Transcription of *gsdmd* is also directly driven by IRF2 for the execution of pyroptosis by binding to a previously uncharacterized but unique site within the *gsdmd* promoter [55,56]. In addition to IRF1/2, IRF8 has been shown to be indispensable for caspase-11-mediated NLRP3 inflammasome activation during LPS transfection [57]. Thus, IRF1/2/8 play critical roles in regulating the expression of inflammatory caspases and GSDMD. In our previous study, we have found that amphioxus contains 9 IRF members [40]. As homologs to vertebrate IRF1 and IRF8, BbIRF1 and BbIRF8 are transcriptional activators that bind to the ISRE motif [40]. By analyzing the promoter sequence of *Bbgsdme*, we found 3 IRF1 or RelA binding sites on its promoter region and demonstrated that these cross-binding motif of NF-κB and IRF1 are important for the transcription of *Bbgsdme*. The cross regulation between NF-κB and IRF in inflammatory responses has attracted much attention in the past decade [58]. Thus, the cross regulation between amphioxus IRF1/8 and p65 may be important for the transcriptional regulation of *Bbgsdme*.

### Diverged GSDM-N generated by different proteolytic processes or alternative splicing may alter the fate of cells

Mammalian GSDMs have been reported to be substrates of CASPs and granzymes, yielding N-termini with distinct sizes and functions. For example, HsGSDMD is cleaved by inflammatory CASPs 1/4/5/11 at D276, which promotes pyroptosis and the release of proinflammatory cytokines [3,4,8]. Additionally, apoptotic CASP8 can cleave HsGSDMD at D276, leading to pyroptosis-like cell death and IL-1β release in murine macrophages [59]. CASP8 can also inactivate HsGSDMD by cleavage at D88, promoting anti-*Yersinia* defense [60,61]. In addition to GSDMD, HsGSDME has been reported to be cleaved by both CASP3 and granzyme B at the same _267_DMPD_270_ site, leading to the enhanced cytotoxicity of GSDME on cancer cells [14,15]. Proteolytic cleavage by proteases can also generate inactive GSDM N-terminal fragments. For example, GSDMD was cleaved into p40-NT fragment through a caspase-independent way, which promoted the secretion of IL-33 without the occurrence of cell death in epithelial cells after exposure to allergen protease [62]. Similar to mammalian GSDMs, amphioxus GSDME can be cleaved by distinct CASPs to generate functionally diverged N-termini. BbGSDME-N253 can form oligomers and directly bind to the inner cell membrane to induce pyroptosis, while BbGSDME-N304 distributed widely in the cytosol can not induce pyroptosis. Since the N304 fragment can be further cleaved by the same BbCASPs into N253 fragment, the generation of N304 and the interaction between N304 and N253 may provide feedback regulation to avoid the excessive inflammation mediated by BbGSDME-N253, just like the feedback regulation of IκB to p65.

Previously, we have found that amphioxus has more than 40 CASPs [35]. By analyzing the EST database, we found that these CASPs are widely distributed and dynamically expressed during embryogenesis in the amphioxus (S8G Fig). Expansion of CASPs was also found in other aquatic species, such as mollusks, echinoderms, and zebrafish [63–65] (S8H Fig), suggesting the complicated activation and regulation of GSDME-mediated pyroptosis in these species. In addition to being processed to produce functionally diverged N-termini, amphioxus GSDME can produce alternative splicing isoforms. Among these isoforms, BbGSDME-S1 encodes a GSEME-N-terminus that can directly cause pyroptosis. This phenomenon was also observed in a few invertebrates by searching the EST database. For example, both *Hydra vulgaris* and *Strongylocentrotus intermedius* have 2 GSDME splicing isoforms, one encoding the FL GSDME homolog and the other containing only the GSDME-N-terminus with potential lytic activity (S1 Table). In addition to BbGSDME-S1, the other BbGSDME isoforms found in amphioxus muscle or skin might be dysfunctional in mediating pyroptosis. Production of active or inactive GSDM-N isoforms through alternative splicing might not be beneficial to the host, as it may induce a continuous inflammatory response and even alter cell survival. Besides, genetic dynamics of GSDME may affect its function and be related with important physiological, as several cancer-related SNP mutations of HsGSDME cause loss-of-function (LOF) [15]. Thus, further investigations on how SNP mutants or splicing isoforms of BbGSDME perform de novo biological functions in amphioxus may be of particular interest for understanding novel functions of GSDMs evolutionarily.

In all, the regulation network of GSDM-mediated inflammation is highly sophisticated in the activation by distinct CASPs or other proteases, the generation of isoforms, and the genetic diversity from our evolutionary study here, which may provide new therapeutic modalities for the treatment of bacterial infection.

## Materials and methods

### Cells

HEK 293T and HeLa cells were purchased from ATCC and preserved in our lab. HeLa^*gsdmd/e DKO*^ cells were kindly presented by Prof.Feng Shao (National Institute of Biological Sciences, Beijing, China) [14]. These cells were cultured in Dulbecco’s modified Eagle’s medium (DMEM) supplemented with 10% fetal bovine serum (FBS; Life Technologies) and antibiotics (streptomycin and penicillin; Life Technologies).

### Microscopy, flow cytometry, and LDH release

To examine the morphology of the pyroptotic cells, HeLa^*gsdmd/e DKO*^ cells were seeded in 12-well plates at 70% confluence, transfected with the indicated plasmids, and treated with 20 ng/ml TNFα and 10 μg/ml CHX for 4 h. Bright-field cell images were captured using a microscope (Leica Microsystems CMS GmbH DMi8). To examine the ratio of pyroptotic cells, treated cells were collected and stained using the annexin V-FITC/PI Apoptosis Assay Kit (Beyotime) according to the manufacturer’s instructions. Stained cells were analyzed by flow cytometry (Beckman Coulter Cyto FLEX S). For the LDH release assays, 293T cells were seeded in a 48-well plate and then transfected with the indicated vectors. After 48 h, cell supernatants were measured using the LDH Cytotoxicity Assay Kit (Beyotime) according to the manufacturer’s instructions.

### Bacterial growth inhibition and protoplast lysis

To assay the cytotoxic effects of BbGSDME and its N-termini in *E*. *coli*, equal numbers of BL21 (DE3) cells were transformed with the indicated expression plasmids. The transformed cells were cultured in Luria-Bertani (LB) medium containing 100 mg/ml ampicillin at 37°C until the OD_600_ reached 1.0. Cells were then diluted and cultured on LB agar plates containing 100 mg/ml ampicillin with or without 0.4 mM IPTG. After incubation at 37°C overnight, the colony-forming units (CFUs) on the plates were counted for statistical analysis.

As for protoplast lysis assays, protoplasts were prepared using a previously reported method [6]. Briefly, *B*. *megaterium* cells were cultured in medium at 30°C until the OD_600_ reached 2.0. After centrifugation, the bacterial pellets were resuspended in buffer (20 mM MgCl_2_, 20 mM sodium malate (pH 6.5), and 500 mM sucrose) and incubated with 2 mg/ml lysozyme at 37°C for 30 min. The formation of protoplasts was examined by microscopy. Before adding the indicated GSDM proteins, the protoplasts were diluted with sucrose buffer until OD_600_ = 1.0. Then, 200 μl of diluted protoplast was added to a 96-well plate and incubated with the indicated GSDM proteins (final concentration of 1 μM) at 37°C for 20 min. To achieve complete lysis of the protoplast, 2% (v/v) Triton X-100 was added as a control. The OD_600_ of the protoplasts incubated with GSDM proteins was measured every 5 min and indicated as A_n_. The OD_600_ of untreated protoplasts was indicated as A_0_. Triton X-100 was used to achieve 100% lysis of the protoplasts and measured as A_100_. The percentage of protoplast lysis was calculated according to the following formula: lysis (%) = (A_0_ − A_n_) × 100 / (A_0_ − A_100_).

### Protein lipid binding assay

Lipid strip (Echelon Biosciences) was first blocked using buffer PBST plus 3% nonfat BSA (Sigma–Aldrich) and gently agitated for 1 h at room temperature (RT). Then, the lipid strip was incubated with 2 μg of the tested proteins and gently agitated for 1 h at RT. After washing 3 times with PBST, the strip was incubated with anti-Flag antibody to detect target proteins that linked a Flag tag.

### Native PAGE gel

Proteins were prepared using a Native PAGE Sample PrepKit (Invitrogen) and electrophoresed with 6% Native PAGE gel in running buffer (10.5 g Bis-Tris, 8.95 g Tricine/1 L) at 4°C and 150 V for 2 h. After transferring proteins to a PVDF membrane at 0.2 A for 1.5 h in transfer buffer (Invitrogen), the membrane was immersed in 8% acetic acid to fix the proteins and then detected by the indicated antibody.

### Dual-luciferase repertory assay

Luciferase repertory assay was conducted as previously reported [39,40]. 293T cells were transfected with HA-tagged BbIRF1/8 and Bbp65 expression plasmids and the indicated reporter vectors, and then relative luciferase activities were detected by the Dual-luciferase reporter assay system (Promega). Renilla luciferase activity was measured and normalized to firefly luciferase activity.

### Bacterial infection of amphioxus and morphology observation

*Edwardsiella tarda* (EIB202) was a gift from Prof. Xuanxian Peng (Sun Yat-sen University, Guangzhou) [66]. EIB202 was cultured in TSB medium at 37°C. The bacteria were harvested by centrifugation, followed by washing and resuspending with PBS. For EIB202 infection experiments, 50 healthy individuals were randomly divided into 5 groups (each group had 10 individuals). Control was cultured in sterile seawater with PBS. The infected group was immersed in seawater with EIB202 (4 × 10^5^ CFU/ml) for 4 h and then cultured in sterile seawater at 26°C. For CASP inhibition, individuals were preincubated with the pan CASP inhibitor (Calbiochem, cot: 627610) or Ac-VHTD-CHO at a final concentration of 1 μM, 2 μM, or 4 μM for 2 h and then immered in EIB202 (4 × 10^5^ CFU/ml) and indicated inhibitor containing seawater. The control group was incubated in PBS containing sterile seawater. The condition of the amphioxus was observed daily. Photos were taken by stereoscopic microscopy (OLYMPUS, SZX16).

### Statistical analysis

Student’s *t*-test was performed to calculate the *P* value between groups using GraphPad Prism (GraphPad Software). Comparisons of survival curves were calculated using the log-rank (Mantel–Cox) test method.

Detailed methods about phylogenetic, exon-intron structure and collinearity analyses, in vitro CASPs cleavage assays, Co-IP assays, western blotting analysis, purification of recombinant proteins from BL21, rabbit antiserum preparation, whole-mount in situ hybridization, RT-PCR analysis, and in vitro bacterial killing assay can be found in S2 Appendix. Details for the reagents and primers used in this study can be found in S4 and S5 Tables.

## Supporting information

S1 Fig An ML phylogenetic tree of GSDM family. Numbers at the nodes of branches represented bootstrap values. Purple arc represents the GSDME/PJVK clade, while the light blue arc stands for the GSDMA/B/C/D clade. The red stars indicated that the member of GSDM family was firstly identified in the evolutionary progress. Dotted arc represents different subgroups of GSDM family. The species abbreviations were listed in S2 Table. GSDM, gasdermin; ML, maximum-likelihood; PJVK, Pejvakin.
(TIF)

S2 Fig The intron phase of *gsdmea*, *gsdmeb*, and *gsdmec* in bony fish. (**A**) MCScanX is used to analyze the gene linkage and collinearity between the *gsdma/b* loci of cartilaginous fish *Callorhinchus milii* and reptile *Podarcis muralis*. The red line indicates the syntenic relationship between *Cmigsdma/b* and *Pmugsdmb*, while the blue line indicates the syntenic relationship between *Pmugsdma* and *Ggagsdma*. (**B**) The intron phases of *gsdmea*, *gsdmeb*, and *gsdmec* in some bony fishes. (**C**) MCScanX is used to analyze the gene linkage and collinearity among *Anguilla anguilla gsdmec*, *C*. *milii gsdme*, and *gsdma/b*.
(TIF)

S3 Fig BbGSDME could convert apoptosis to pyroptosis in HeLa upon TNFα plus CHX treatment. (**A**) One of the presentative results of flow-cytometric analysis of annexin V-FITC and/or PI staining in HeLa^*gsdmd/e DKO*^ cells upon indicated transfection and treatments. (**B**, **C**) Percentage of Annexin V–positive alone cells (**B**) and PI-positive alone cells (**C**) upon indicated transfection and treatments. (**D**) The effect of indicated GSDME homologs on IL-6 release in HeLa^*gsdmd/e DKO*^ cells with or without treatments of TNFα plus CHX. The release of IL-6 was detected by ELISA. Data represent the mean ± SD of 3 independent experiments. *P* values were analyzed with Student’s *t*-test. (**E**) Cell morphological images of HeLa cells, which were transfected with the BbGSDME and its mutants constructs and then stimulated with TNFα plus CHX treatment for 4 h. (**F**) The potential CASP cleavage motif in GSDME among species. Tetrapeptide motifs were drawn by WebLogo analysis. P4-P1 and P1’ represented the contiguous amino acids for the recognition by CASPs. (**G**) The distribution of DVVD/VHTD motifs in other GSDM proteins. The species abbreviations were listed in S2 Table. All FCS files are available from the FlowRepository database (Repository ID: FR-FCM-Z642). Full gating strategies from representative plots are shown in S1 Gating Strategy. Raw data can be found in Supporting information (S1 Values For Plots). CHX, cycloheximide; GSDM, gasdermin; IL-6, interlukin 6; PI, propidium iodide; TNFα, tumor necrosis factor alpha.
(TIF)

S4 Fig The alternative splicing and SNPs of *Bbgsdme*. (**A**, **B**) The structure surface of BbGSDME-N304 interdomain interfaces (**A**) and charge distribution in BbGSDME-N304 (**B**). (**C**) Co-IP analyses showed that BbGSDME-N253 could interact with BbGSDME-N304, but not BbGSDME-FL. (**D**) Co-IP analyses showed that BbGSDME-N304 could be self-associated but not interact with BbGSDME-FL. (**E**) RT-PCR analyses indicated that *Bbgsdme* has distinct splicing isoforms. Numbers indicated distinct amphioxus individuals. At right, RT-PCR analyses using tissues from the same amphioxus adult identified 5 more *Bbgsdme* splicing variants. S1-S6 represent different alternative splicing isoforms. (**F**) The schematic diagram of distinct *Bbgsdme* splicing isoforms indicated in (**E**). The genome sequence (NW_017804675.1) coding for *Bbgsdme* was obtained from NCBI. Colored rectangles indicated exons. (**G**) LDH release mediated by BbGSDME-FL, BbGSDME-S1, and BbGSDMES2 in 293T cells. *n =* 3, Student’s *t*-test. (**H**) BbGSDME-S1 but not BbGSDME-S2 could induce pyroptosis in 293T cells. Cell morphological images shown were representative of 3 independent biological experiments. Raw data can be found in Supporting information (S1 Raw Images and S1 Values For Plots files). G, gill; H, hepatic caecum; I, intestines; LDH, lactate dehydrogenase; M, muscle; RT-PCR, reverse transcription PCR; S, skin; SNP, single nucleotide polymorphism; WCL, whole cell lysate.
(TIF)

S5 Fig The alignment of the protein sequences of BbGSDME splicing isoforms. Conserved residues were colored in red. Blue box highlighted the sequences with similarity. Figure is shown by ENDscript.
(TIF)

S6 Fig The expression pattern of *Bbgsdme* and its relation with bacterial infection. (**A**) The expression of *Bbgsdme* in amphioxus embryos. Whole-mount in situ hybridization of *Branchiostoma floridae* embryos. The top shows different stages of embryos. 5S, 8S indicate embryos in 5 somite and 8 somite stages during neurula development. Scale bar is 100 μm. (**B**) Distribution of *Bbgsdme* in various tissues was determined using qRT-PCR analysis. The transcription of *Bbgsdme* in hepatic cecum was set to 1 to calculate the relative expression. Data represent the mean ± SD of 3 independent experiments. (**C**) Images of injured *Branchiostoma belcheri* in widefield. I, II, III indicate 3 kinds of progressive damage degrees. Scale bar is 1 mm. (**D**) CBB staining and WB were used to detect the purity of the purified BbGSDME protein from BL21. (**E**) WB assays to detect the specificity and titer of the antiserum against BbGSDME using the Flag-tagged BbGSDME, which were overexpressed in 293T cells. (**F**) WB assays to detect the cross-reaction of anti-BbGSDME serum using the indicated GSDM homologs, which were overexpressed in 293T cells. (**G**) Surviving curve for amphioxus challenged with or without EIB202 at the presence of Z-VAD-FMK. *P* values were calculated by the log-rank (Mantel–Cox) test method. (**H**) The morphology of amphioxus individuals, which were infected with EIB202 (4 × 10^5^ CFU/ml, on day 3 as showed in **G**). Black scale bar is 1 cm; white scale bar is 500 μm. (**I**) Ac-VHTD-CHO inhibited the cleavage of HsGSDME-VHTD by the active form of rHsCASP3 in vitro. (**J**) The number of proteins containing the DVVD or VHTD motif in amphioxus or the DMPD motif in humans. More details are shown in S3 Table. (**K**) The dual-luciferase reporter assays indicated that Bbp65 can induce the expression of reporter in HEK293T cells. Data represent the mean ± SD of 3 independent experiments. *P* values were analyzed with Student’s *t*-test. Raw data can be found in Supporting information (S1 Raw Images and S1 Values For Plots files). CBB, Coomassie brilliant blue; CFU, colony-forming unit; G, gill; GSDM, gasdermin; H, hepatic caecum; I, intestines; LN, late neurula; M, muscle; qRT-PCR, quantitative real time PCR; S, skin; WB, western blot.
(TIF)

S7 Fig Functional alteration of HsGSDME and the genetic diversity of BbGSDME. (**A**) The predicted 3D structure of BbGSDME by comparing with HsGSDMD (PDB: 6vfe.1). The homology model for BbGSDME was generated by SWISS-MODEL server. The model diagrams were prepared by UCSF Chimera. (**B**) Pyroptosis morphology of HeLa^*gsdmd/e DKO*^ cells, which were transfected with HsGSDME and its NT mutants. Scale bar, 75 μm. Representative images from 3 independent biological experiments were shown. (**C**) Multiple sequence alignment of the GSDM members. The alignment was performed using MAFFT. Numbers indicated the residues of HsGSDME. (**D**) Pyroptosis morphology of 293T cells, which were transfected with HsGSDMD-NT and its mutants. Scale bar, 75 μm. Representative images from 3 independent biological experiments were shown. (**E**) Overall structure of HsGSDMD-NT (PDB: 6vfe) was drawn by UCSF. Residues S212-T213 and L16 are located at the interface of GSDMD-NT subunits. Blue and gray indicate adjacent subunits. (**F**) The SNPs of HsGSDME were observed from the ClinVar database. Red color highlights the SNPs that can affect the activity of HsGSDME. (**G**) The pyroptotic activity of HsGSDME-NT and its mutants. Indicated HsGSDME constructs were expressed in HeLa^*gsdmd/e DKO*^ cells for 48 h. *n =* 3, Student’s *t*-test. (**H**) Cell morphology of HeLa^*gsdmd/e DKO*^ cells, which were transfected with wild-type HsGSDME-NT or HsGSDME-NT mutants. Scale bar, 50 μm. All cell morphological images shown were representative of 3 independent biological experiments. (**I**) The SNP analysis of 8 distinct BbGSDME-FL that were obtained from 8 distinct amphioxus individuals. Alignment was done by ClusterW and analyzed by DIVEIN. (**J**) The nonsynonymous mutations may alter the function of *Bbgsdme*. (**K**, **L**) The nonsynonymous mutations, which may alter the lipids binding activity and oligomerization activity of BbGSDME, were indicated in the predicted 3D models. Raw data can be found in Supporting information (S1 Values For Plots). GSDM, gasdermin; SNP, single nucleotide polymorphism.
(TIF)

S8 Fig The domain architectures of NLRs, ASCs, and CASPs in amphioxus and human, and the expression pattern of PJVKs and CASPs. (**A**) BbGSDME-N253 could not lyse EIB202. Dilution series of EIB202 in PBS were treated with indicated GSDM proteins. After incubation at 37°C for 2 h, reactions were diluted in 10-fold increments and plated on TSB agar. (**B**, **C**) LPS immersion could not induce the cleavage of BbGSDME-N253 in adult amphioxus. (**D**) EIB202 labeled by GFP could invade HeLa cells. EIB202 (1 × 10^7^ CFU/ml) were incubated with HeLa cells for 8 h. Scale bar, 25 μm. (**E**) The domain architectures of NLRs, ASCs, and CASPs in amphioxus and human. (**F**) The expression profiles of HsPJVK and DrPJVK were obtained from BioProject PRJEB4337 and PRJNA255848, respectively. (**G**) The expression profile of amphioxus CASPs during embryogenesis was obtained from lncDNA-BF (http://139.129.29.118/IncDNA/index.jsp). (**H**) The expression profile of zebrafish CASPs in distinct tissues was obtained from BioProject PRJNA255848. Raw data can be found in Supporting information (S1 Raw Images and S1 Values For Plots files). CRAD, caspase recruitment domain; DD, death domain; DED, death effector domain; GSDM, gasdermin; LPS, lipopolysaccharide; LRR, leucine-rich repeat; PJVK, Pejvakin; PYD, N-terminal pyrin domain; TSB, trypticase soy broth.
(TIF)

S1 Table The composition of GSDM members among species. The protein sequences were obtained from NCBI Ensemble or UCSC Genome Browser (http://www.genome.ucsc.edu/). The domain architectures were predicted by Pfam (http://pfam.xfam.org/) program. White rectangle indicates the GSDM pore-forming domain, and black rectangle stands for GSDM PUB domain. Black star indicates the GSDM homologs first emerged in distinct evolutionary stages. E-L indicates the FL GSDME. E-S, E-S1, E-S2 et al indicate the GSDME alternative splicing isoforms. Ea and Eb indicate distinct GSDME genes arrayed on the same scaffold. A to A6 or C to C6 indicate the expanded GSDMA and GSDMC genes in specific species, respectively. GSDM, gasdermin; NCBI, The National Center for Biotechnology Information; PJVK, Pejvakin.
(PDF)

S2 Table The intron phases of GSDM members among species. The Latin names of species and the accession numbers of GSDM proteins used in the phylogenetic and intron phase analyses were listed in this excel table. The numbers 0, 1, 2 indicate the intron phases of specific genes. S2 Table was submitted as a separated excel file.
(XLSX)

S3 Table The accession number of the proteins containing DVVD or VHTD in amphioxus and DMPD in humans. Accession numbers labeled by red indicate the proteins containing the VHTD A/G/S motif in amphioxus.
(XLSX)

S4 Table Reagents, antibodies, cells, and microbes used in this study. (PDF)

S5 Table Primers used for RT-PCR in this study. (PDF)

S1 Appendix The sequence alignments of GSDME and PJVK with secondary structure elements. (PDF)

S2 Appendix Supplementary materials and methods. (PDF)

S1 Raw Images Original scan images for Figs 2E–2G, 2J, 2K, 2M–2O, 3E, 3H–3I, 4A–4E, 4I, 4O–4P, 4E, 5J, S4C–S4E, S6D–S6F, S6I and S8B–S8C. ‘✕’ indicates irrelevant samples or irrelevant membranes. (PDF)

S1 Values For Plots Numerical values used for plots and statistical analysis in Figs 2C–2D, 2H, 3B–3E, 3L, 4F, 4L-4M, 5B–5D, 5G–5I, 5K–5L, S3B–S3D, S4G, S6B, S6G, S6K, S7G, and S8F–S8H. (XLSX)

S1 Gating Strategy Full gating strategy from representative plots shown in Figs 2C and S3A–S3C. (PDF)

## Abbreviations

Bb: *Branchiostoma belcheri*
CASP1: caspase-1
CASP3: caspase 3
CHX: cycloheximide
DED: death effector domain
Dr: *Danio rerio*
EST: expressed sequence tag
FL: full-length
GAS: group A *Streptococcus*
GSDM: gasdermin
HE: hematoxylin–eosin
IL-1: interleukin-1
IL-6: interlukin 6
IPTG: isopropyl β-D-thiogalactoside
IRF1: interferon regulatory factor 1
ISRE: IFN-stimulated response element
κB: kappa B
LB: Luria-Bertani
LDH: lactate dehydrogenase
LOF: loss-of-function
LPS: lipopolysaccharide
ML: maximum-likelihood
PI: propidium iodide
PJVK: Pejvakin
RT: room temperature
Rcd-1: regulator cell death
SNP: single nucleotide polymorphism
SpeB: *streptococcal pyrogenic* exotoxin B
TNFα: tumor necrosis factor alpha
WB: western blot
WCL: whole cell lysate
