# Supplementary figures and images for: Characterization of GSDME in amphioxus provides insights into the functional evolution of GSDM-mediated pyroptosis
Source: PLoS Biol. 2023 May 3;21(5):e3002062. doi: 10.1371/journal.pbio.3002062 (PMC10155998; doi:10.1371/journal.pbio.3002062)

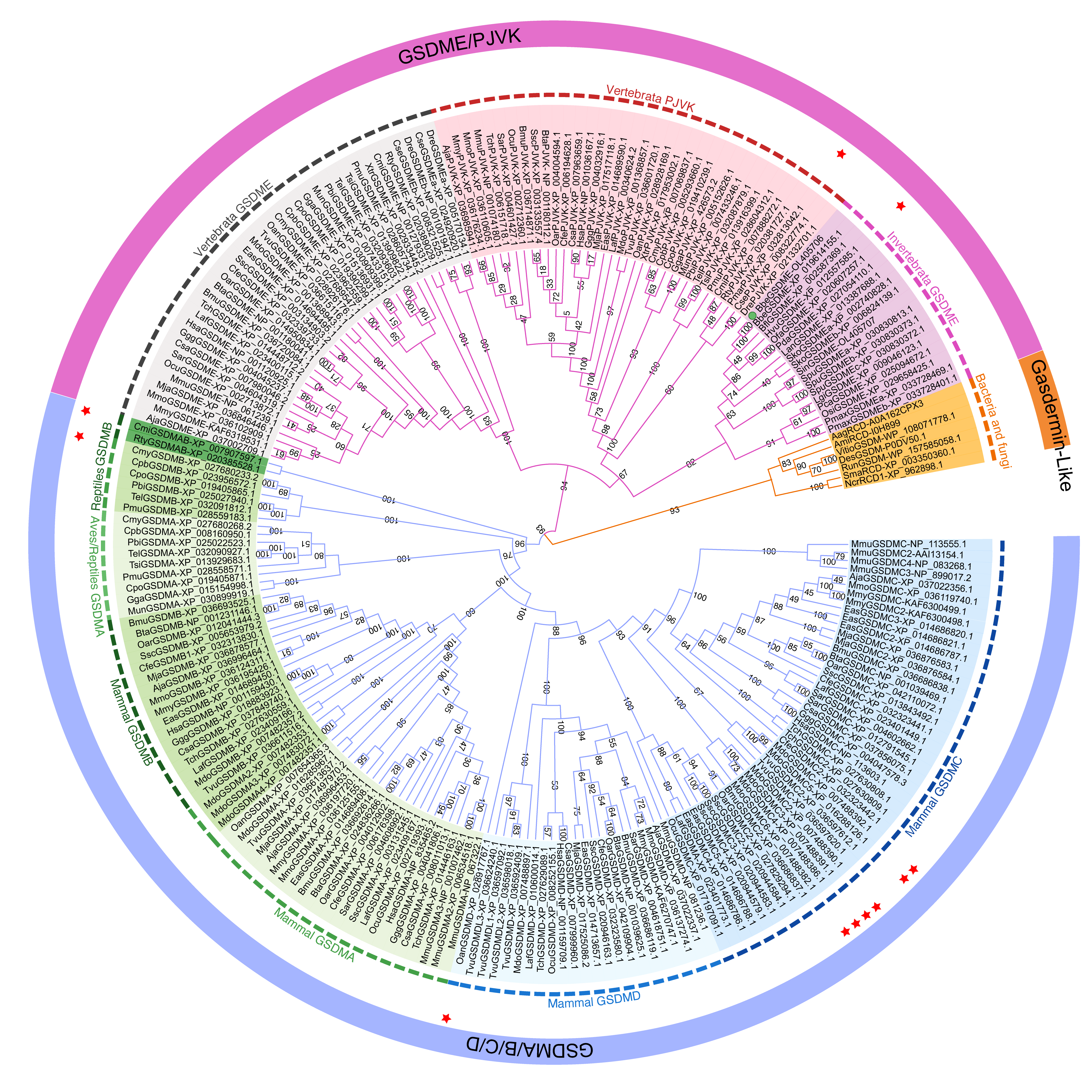

Supplement: S1 Fig — Numbers at the nodes of branches represented bootstrap values. Purple arc represents the GSDME/PJVK clade, while the light blue arc stands for the GSDMA/B/C/D clade. The red stars indicated that the member of GSDM family was firstly identified in the evolutionary progress. Dotted arc represents different subgroups of GSDM family. The species abbreviations were listed in S2 Table. GSDM, gasdermin; ML, maximum-likelihood; PJVK, Pejvakin. (TIF) [file pbio.3002062.s001.tif]

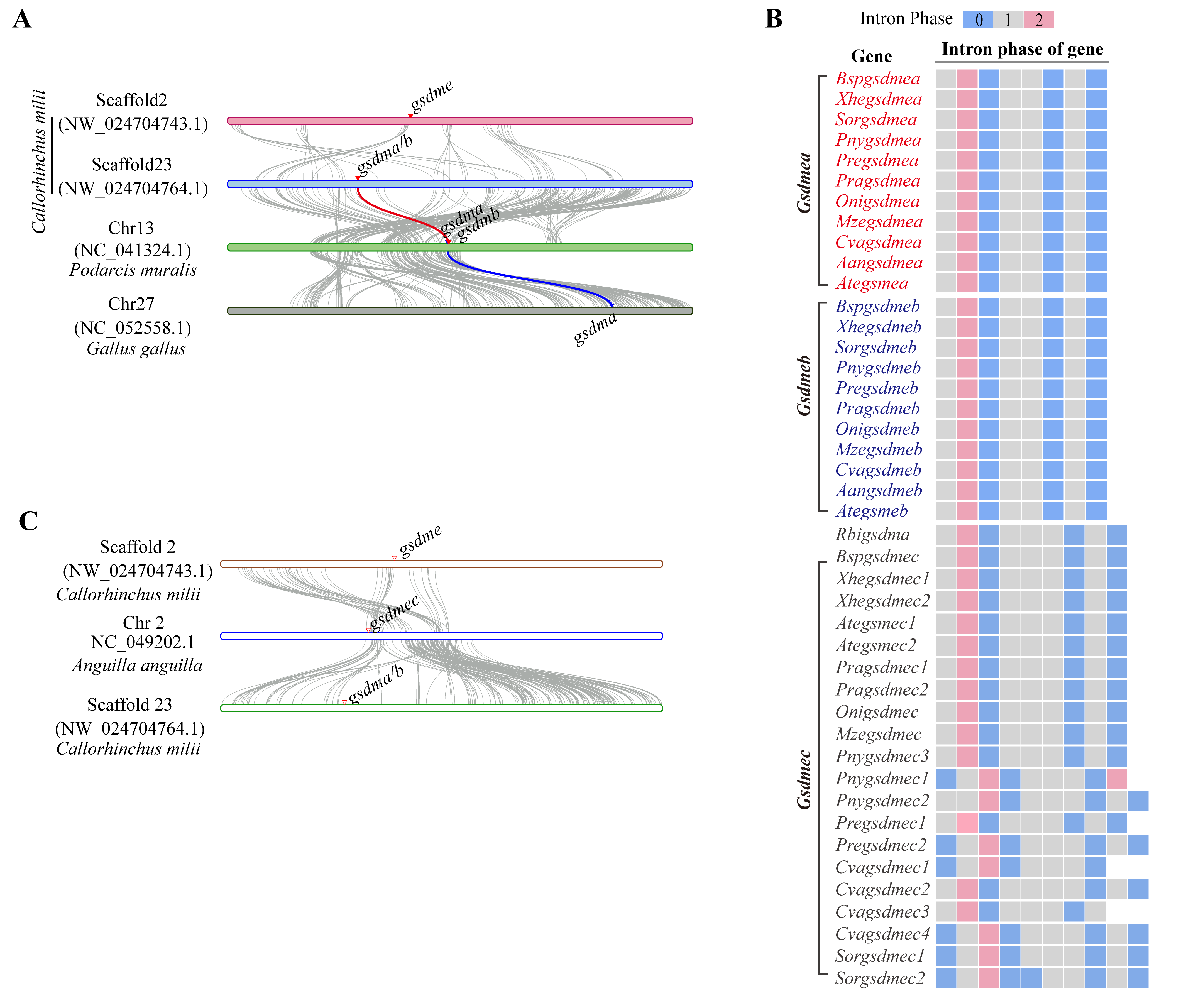

Supplement: S2 Fig — (A) MCScanX is used to analyze the gene linkage and collinearity between the gsdma/b loci of cartilaginous fish Callorhinchus milii and reptile Podarcis muralis. The red line indicates the syntenic relationship between Cmigsdma/b and Pmugsdmb, while the blue line indicates the syntenic relationship between Pmugsdma and Ggagsdma. (B) The intron phases of gsdmea, gsdmeb, and gsdmec in some bony fishes. (C) MCScanX is used to analyze the gene linkage and collinearity among Anguilla anguilla gsdmec, C. milii gsdme, and gsdma/b. (TIF) [file pbio.3002062.s002.tif]

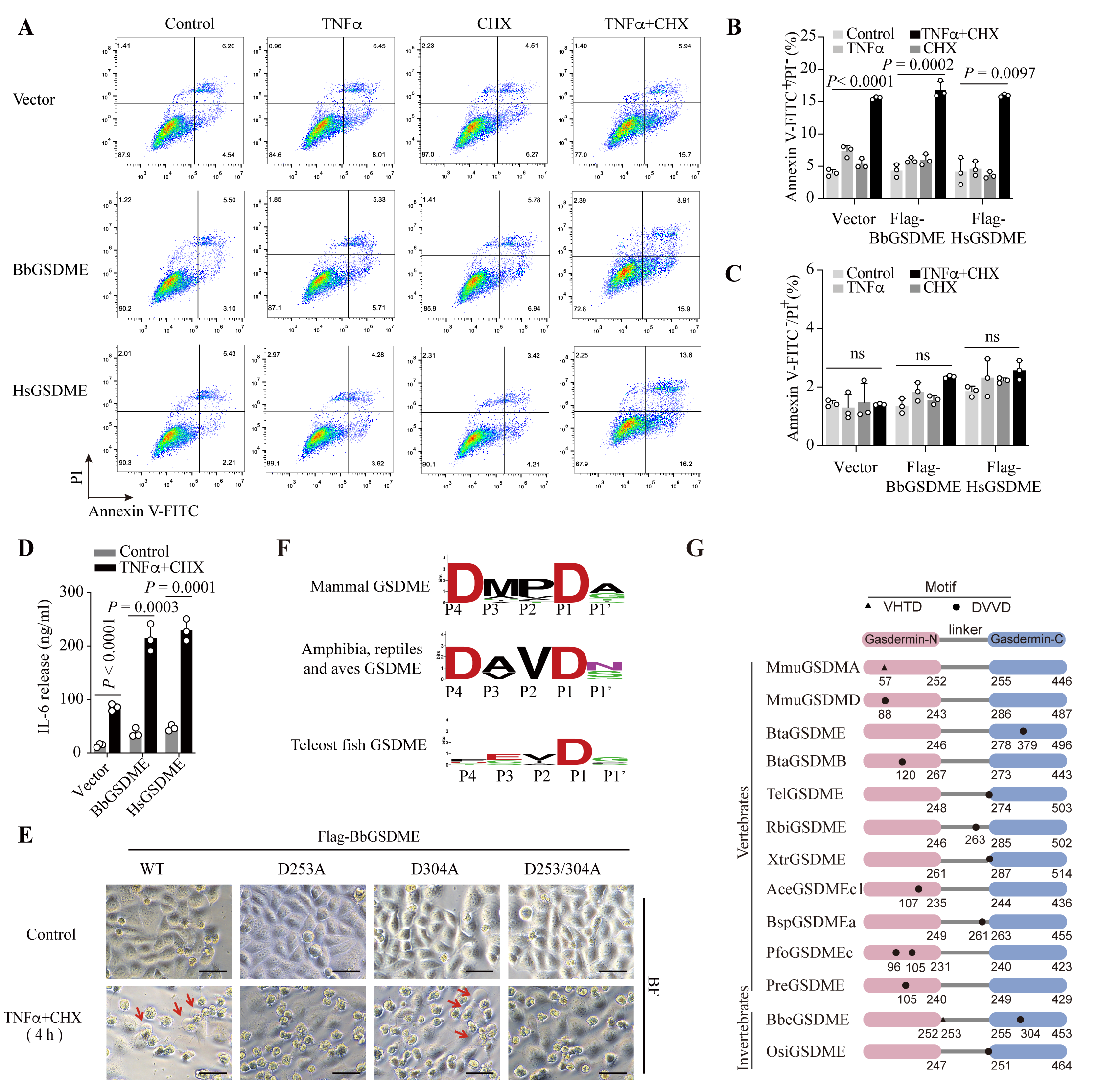

Supplement: S3 Fig — (A) One of the presentative results of flow-cytometric analysis of annexin V-FITC and/or PI staining in HeLagsdmd/e DKO cells upon indicated transfection and treatments. (B, C) Percentage of Annexin V–positive alone cells (B) and PI-positive alone cells (C) upon indicated transfection and treatments. (D) The effect of indicated GSDME homologs on IL-6 release in HeLagsdmd/e DKO cells with or without treatments of TNFα plus CHX. The release of IL-6 was detected by ELISA. Data represent the mean ± SD of 3 independent experiments. P values were analyzed with Student’s t-test. (E) Cell morphological images of HeLa cells, which were transfected with the BbGSDME and its mutants constructs and then stimulated with TNFα plus CHX treatment for 4 h. (F) The potential CASP cleavage motif in GSDME among species. Tetrapeptide motifs were drawn by WebLogo analysis. P4-P1 and P1’ represented the contiguous amino acids for the recognition by CASPs. (G) The distribution of DVVD/VHTD motifs in other GSDM proteins. The species abbreviations were listed in S2 Table. All FCS files are available from the FlowRepository database (Repository ID: FR-FCM-Z642). Full gating strategies from representative plots are shown in S1 Gating Strategy. Raw data can be found in Supporting information (S1 Values For Plots). CHX, cycloheximide; GSDM, gasdermin; IL-6, interlukin 6; PI, propidium iodide; TNFα, tumor necrosis factor alpha. (TIF) [file pbio.3002062.s003.tif]

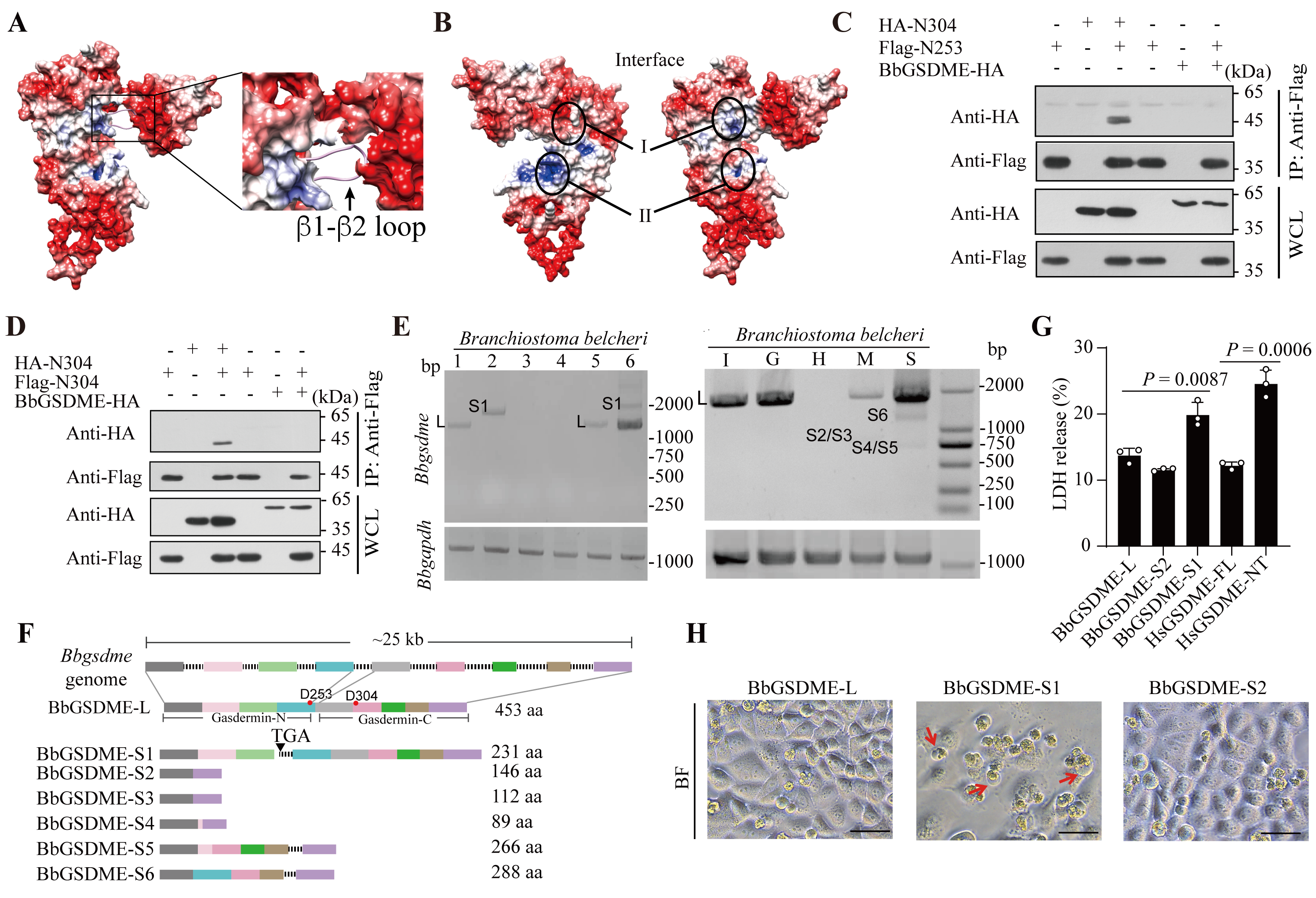

Supplement: S4 Fig — (A, B) The structure surface of BbGSDME-N304 interdomain interfaces (A) and charge distribution in BbGSDME-N304 (B). (C) Co-IP analyses showed that BbGSDME-N253 could interact with BbGSDME-N304, but not BbGSDME-FL. (D) Co-IP analyses showed that BbGSDME-N304 could be self-associated but not interact with BbGSDME-FL. (E) RT-PCR analyses indicated that Bbgsdme has distinct splicing isoforms. Numbers indicated distinct amphioxus individuals. At right, RT-PCR analyses using tissues from the same amphioxus adult identified 5 more Bbgsdme splicing variants. S1-S6 represent different alternative splicing isoforms. (F) The schematic diagram of distinct Bbgsdme splicing isoforms indicated in (E). The genome sequence (NW_017804675.1) coding for Bbgsdme was obtained from NCBI. Colored rectangles indicated exons. (G) LDH release mediated by BbGSDME-FL, BbGSDME-S1, and BbGSDMES2 in 293T cells. n = 3, Student’s t-test. (H) BbGSDME-S1 but not BbGSDME-S2 could induce pyroptosis in 293T cells. Cell morphological images shown were representative of 3 independent biological experiments. Raw data can be found in Supporting information (S1 Raw Images and S1 Values For Plots files). G, gill; H, hepatic caecum; I, intestines; LDH, lactate dehydrogenase; M, muscle; RT-PCR, reverse transcription PCR; S, skin; SNP, single nucleotide polymorphism; WCL, whole cell lysate. (TIF) [file pbio.3002062.s004.tif]

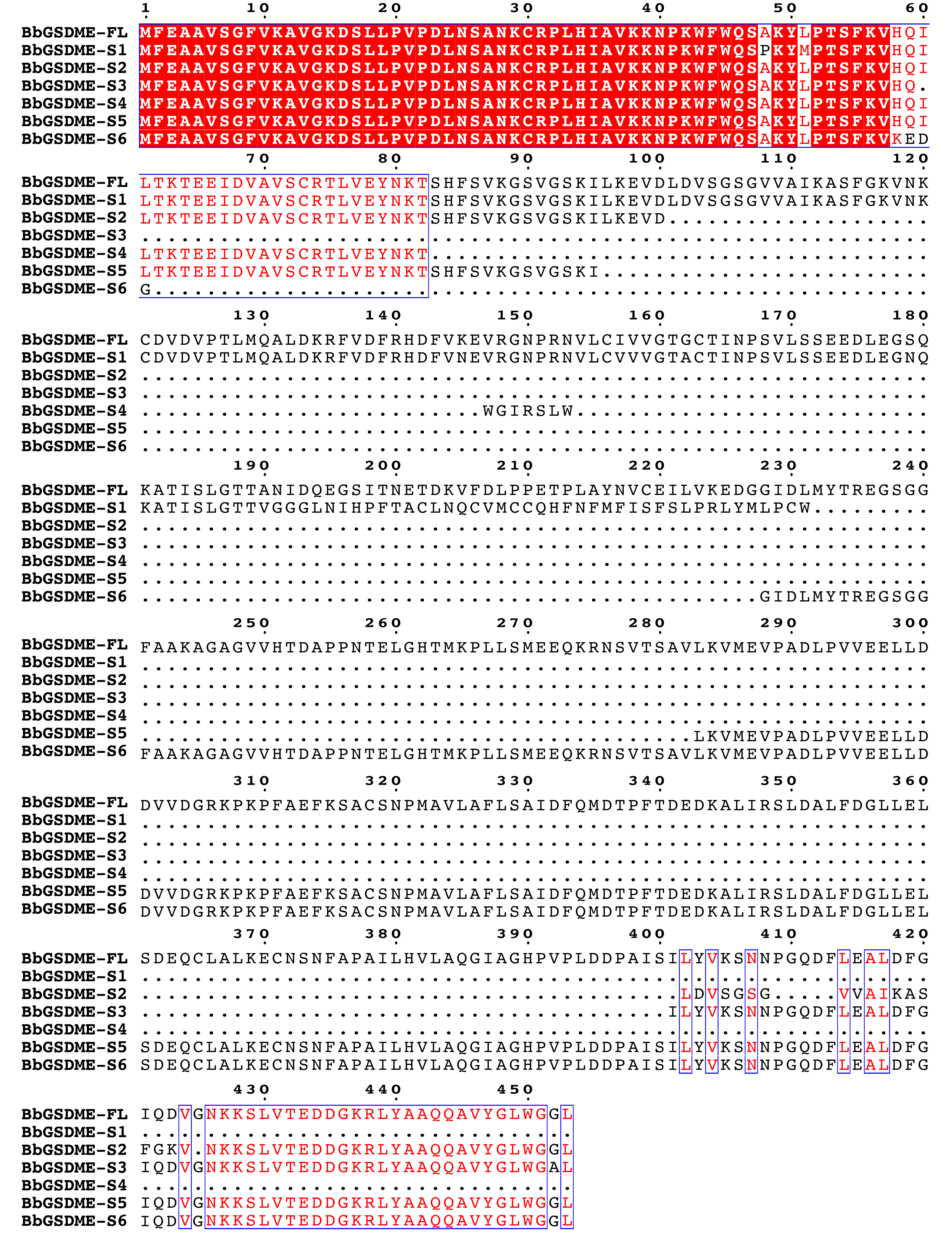

Supplement: S5 Fig — Conserved residues were colored in red. Blue box highlighted the sequences with similarity. Figure is shown by ENDscript. (TIF) [file pbio.3002062.s005.tif]

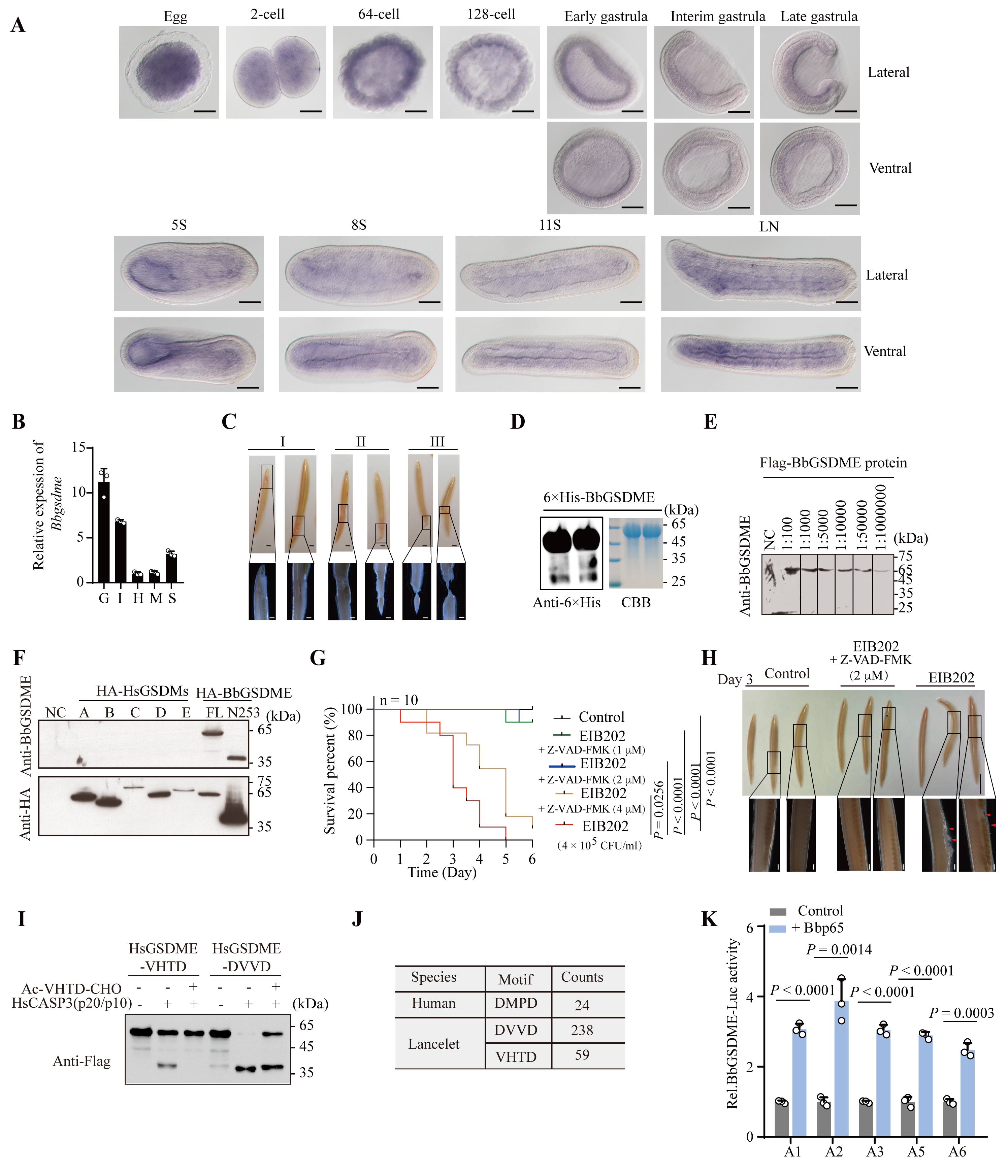

Supplement: S6 Fig — (A) The expression of Bbgsdme in amphioxus embryos. Whole-mount in situ hybridization of Branchiostoma floridae embryos. The top shows different stages of embryos. 5S, 8S indicate embryos in 5 somite and 8 somite stages during neurula development. Scale bar is 100 μm. (B) Distribution of Bbgsdme in various tissues was determined using qRT-PCR analysis. The transcription of Bbgsdme in hepatic cecum was set to 1 to calculate the relative expression. Data represent the mean ± SD of 3 independent experiments. (C) Images of injured Branchiostoma belcheri in widefield. I, II, III indicate 3 kinds of progressive damage degrees. Scale bar is 1 mm. (D) CBB staining and WB were used to detect the purity of the purified BbGSDME protein from BL21. (E) WB assays to detect the specificity and titer of the antiserum against BbGSDME using the Flag-tagged BbGSDME, which were overexpressed in 293T cells. (F) WB assays to detect the cross-reaction of anti-BbGSDME serum using the indicated GSDM homologs, which were overexpressed in 293T cells. (G) Surviving curve for amphioxus challenged with or without EIB202 at the presence of Z-VAD-FMK. P values were calculated by the log-rank (Mantel–Cox) test method. (H) The morphology of amphioxus individuals, which were infected with EIB202 (4 × 10^5^ CFU/ml, on day 3 as showed in G). Black scale bar is 1 cm; white scale bar is 500 μm. (I) Ac-VHTD-CHO inhibited the cleavage of HsGSDME-VHTD by the active form of rHsCASP3 in vitro. (J) The number of proteins containing the DVVD or VHTD motif in amphioxus or the DMPD motif in humans. More details are shown in S3 Table. (K) The dual-luciferase reporter assays indicated that Bbp65 can induce the expression of reporter in HEK293T cells. Data represent the mean ± SD of 3 independent experiments. P values were analyzed with Student’s t-test. Raw data can be found in Supporting information (S1 Raw Images and S1 Values For Plots files). CBB, Coomassie brilliant blue; CFU, colony-forming unit; G, gill; GSDM, [file pbio.3002062.s006.tif]

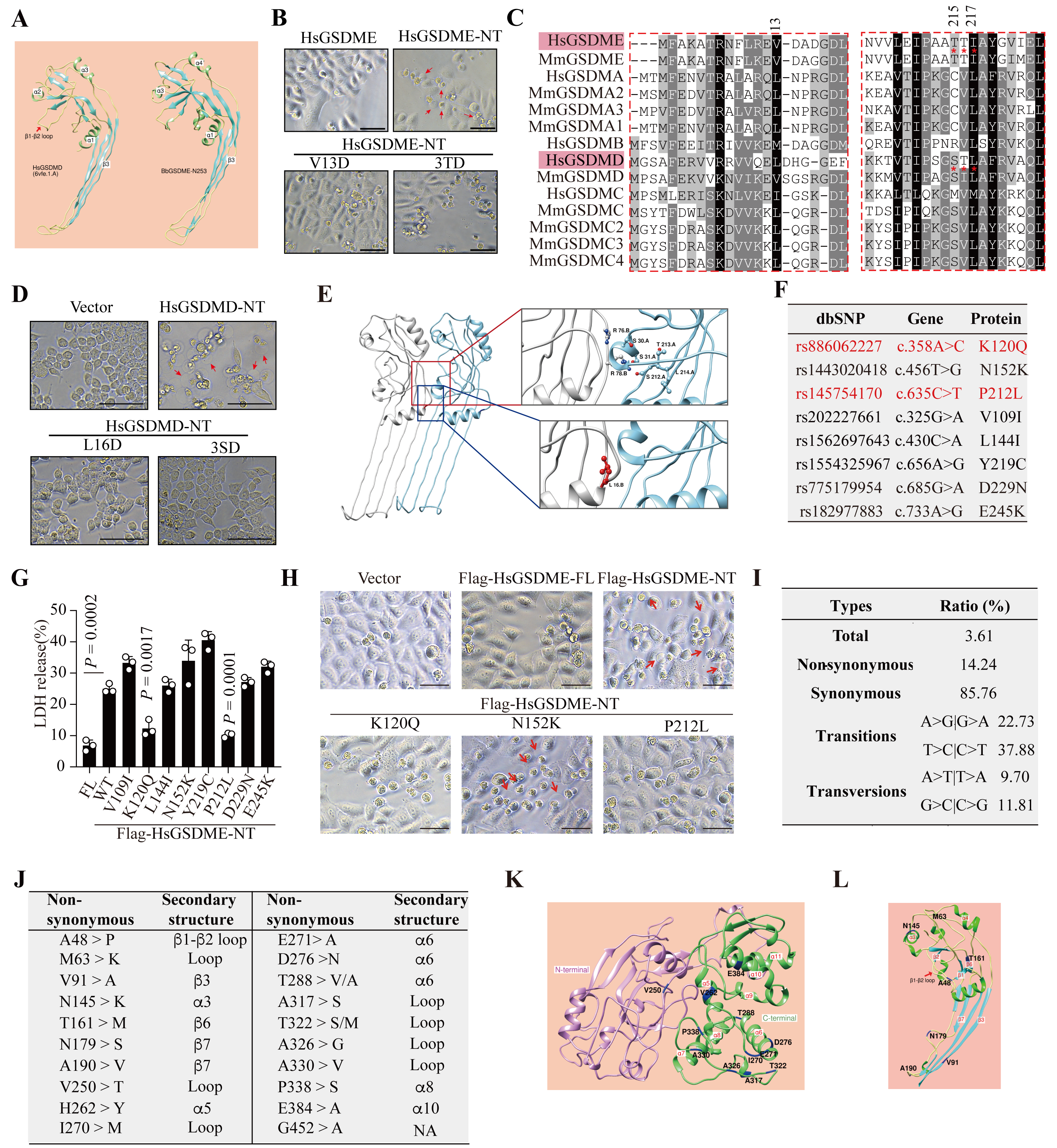

Supplement: S7 Fig — (A) The predicted 3D structure of BbGSDME by comparing with HsGSDMD (PDB: 6vfe.1). The homology model for BbGSDME was generated by SWISS-MODEL server. The model diagrams were prepared by UCSF Chimera. (B) Pyroptosis morphology of HeLagsdmd/e DKO cells, which were transfected with HsGSDME and its NT mutants. Scale bar, 75 μm. Representative images from 3 independent biological experiments were shown. (C) Multiple sequence alignment of the GSDM members. The alignment was performed using MAFFT. Numbers indicated the residues of HsGSDME. (D) Pyroptosis morphology of 293T cells, which were transfected with HsGSDMD-NT and its mutants. Scale bar, 75 μm. Representative images from 3 independent biological experiments were shown. (E) Overall structure of HsGSDMD-NT (PDB: 6vfe) was drawn by UCSF. Residues S212-T213 and L16 are located at the interface of GSDMD-NT subunits. Blue and gray indicate adjacent subunits. (F) The SNPs of HsGSDME were observed from the ClinVar database. Red color highlights the SNPs that can affect the activity of HsGSDME. (G) The pyroptotic activity of HsGSDME-NT and its mutants. Indicated HsGSDME constructs were expressed in HeLagsdmd/e DKO cells for 48 h. n = 3, Student’s t-test. (H) Cell morphology of HeLagsdmd/e DKO cells, which were transfected with wild-type HsGSDME-NT or HsGSDME-NT mutants. Scale bar, 50 μm. All cell morphological images shown were representative of 3 independent biological experiments. (I) The SNP analysis of 8 distinct BbGSDME-FL that were obtained from 8 distinct amphioxus individuals. Alignment was done by ClusterW and analyzed by DIVEIN. (J) The nonsynonymous mutations may alter the function of Bbgsdme. (K, L) The nonsynonymous mutations, which may alter the lipids binding activity and oligomerization activity of BbGSDME, were indicated in the predicted 3D models. Raw data can be found in Supporting information (S1 Values For Plots). GSDM, gasdermin; SNP, single nucleotide polymorphism. (TIF) [file pbio.3002062.s007.tif]

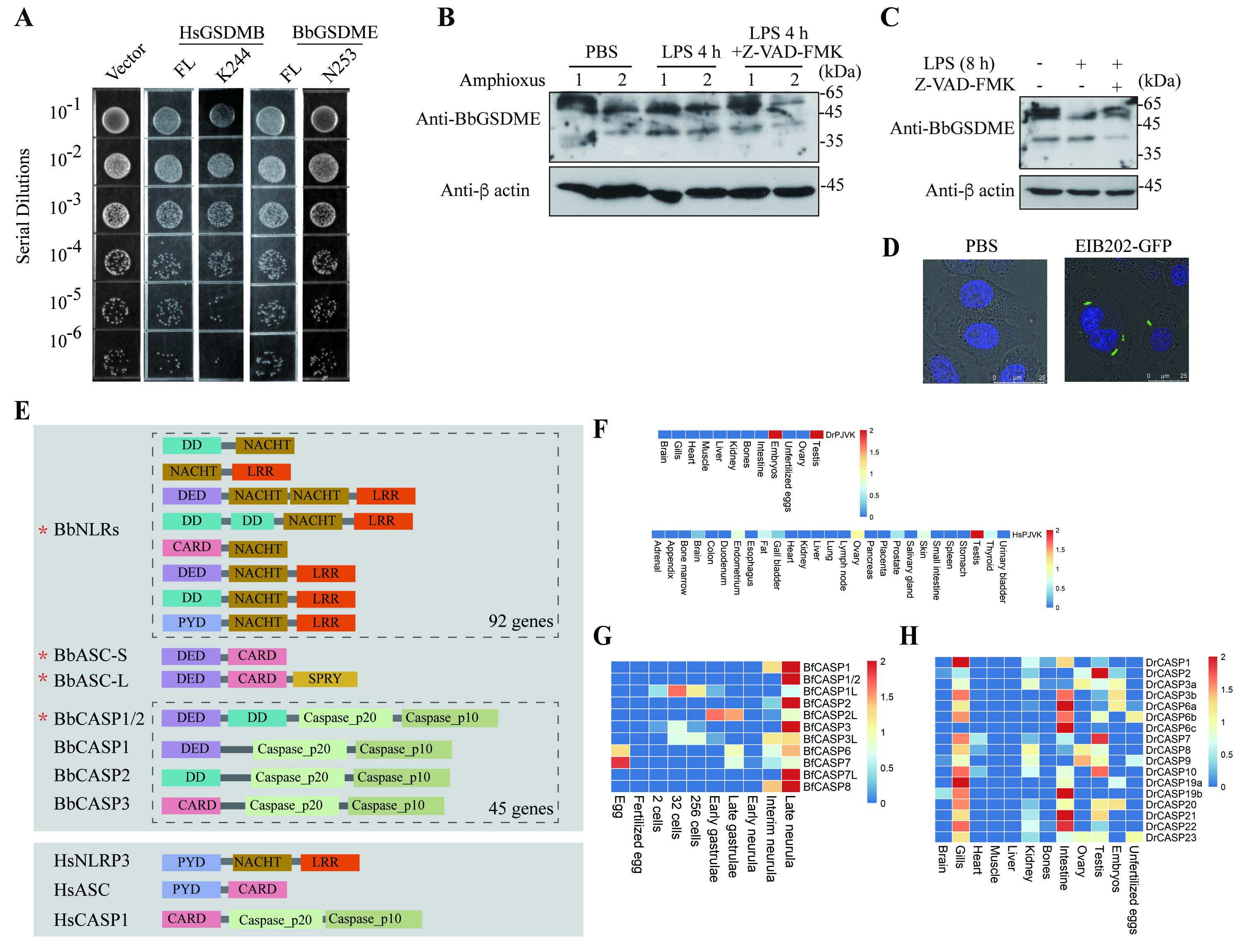

Supplement: S8 Fig — (A) BbGSDME-N253 could not lyse EIB202. Dilution series of EIB202 in PBS were treated with indicated GSDM proteins. After incubation at 37°C for 2 h, reactions were diluted in 10-fold increments and plated on TSB agar. (B, C) LPS immersion could not induce the cleavage of BbGSDME-N253 in adult amphioxus. (D) EIB202 labeled by GFP could invade HeLa cells. EIB202 (1 × 10^7^ CFU/ml) were incubated with HeLa cells for 8 h. Scale bar, 25 μm. (E) The domain architectures of NLRs, ASCs, and CASPs in amphioxus and human. (F) The expression profiles of HsPJVK and DrPJVK were obtained from BioProject PRJEB4337 and PRJNA255848, respectively. (G) The expression profile of amphioxus CASPs during embryogenesis was obtained from lncDNA-BF (http://139.129.29.118/IncDNA/index.jsp). (H) The expression profile of zebrafish CASPs in distinct tissues was obtained from BioProject PRJNA255848. Raw data can be found in Supporting information (S1 Raw Images and S1 Values For Plots files). CRAD, caspase recruitment domain; DD, death domain; DED, death effector domain; GSDM, gasdermin; LPS, lipopolysaccharide; LRR, leucine-rich repeat; PJVK, Pejvakin; PYD, N-terminal pyrin domain; TSB, trypticase soy broth. (TIF) [file pbio.3002062.s008.tif]

Annexin V-FITC and PI staining assay gating strategy used in the following figures: FigS3A

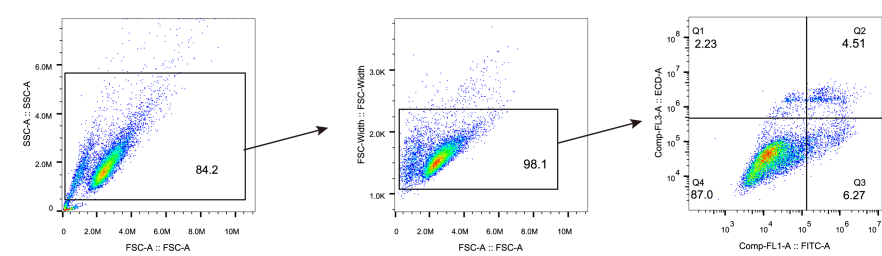

Supplement: S1 Gating Strategy — (PDF) [file pbio.3002062.s018.pdf]
