# Supplementary material for: Characterization of GSDME in amphioxus provides insights into the functional evolution of GSDM-mediated pyroptosis
Source: PLoS Biol. 2023 May 3;21(5):e3002062. doi: 10.1371/journal.pbio.3002062 (PMC10155998; doi:10.1371/journal.pbio.3002062)
Supplement: S1 Table — The protein sequences were obtained from NCBI Ensemble or UCSC Genome Browser (http://www.genome.ucsc.edu/). The domain architectures were predicted by Pfam (http://pfam.xfam.org/) program. White rectangle indicates the GSDM pore-forming domain, and black rectangle stands for GSDM PUB domain. Black star indicates the GSDM homologs first emerged in distinct evolutionary stages. E-L indicates the FL GSDME. E-S, E-S1, E-S2 et al indicate the GSDME alternative splicing isoforms. Ea and Eb indicate distinct GSDME genes arrayed on the same scaffold. A to A6 or C to C6 indicate the expanded GSDMA and GSDMC genes in specific species, respectively. GSDM, gasdermin; NCBI, The National Center for Biotechnology Information; PJVK, Pejvakin. (PDF) [file pbio.3002062.s009.pdf]

S1 Table. The composition of GSDM members among species

Species	PJVK	GSDME	GSDMA	GSDMB	GSDMC	GSDMD
Bacteria	Gasdermin-like					
Fungi	Rcd-1		Het-Q1			
Hydra vulgaris		E-L ★<br>E-S				
Orbicella faveolata						
Nematostella vectensi						
Pecten maximus		Ea<br>Eb				
Saccoglossus kowalevskii		Ea<br>Eb				
Strongylocentrotus purpuratus		Ea<br>Eb<br>Ec				
Strongylocentrotus intermedius		E-L<br>E-S				
Branchiostoma belcheri		E-L<br>E-S1 ……				
Petromyzon marinus	★					
Callorhinchus milii			★			
Danio rerio		Ea<br>Eb				
Anguilla anguilla		Ea<br>Eb	Ec			
Xenopus tropicalis						
Rhinatrema bivittatum			★			
Thamnophis sirtalis						
Podarcis muralis				★		
Gallus gallus						
Ornithorhynchus anatinus						★
Monodelphis domestica			A<br>A2<br>A3<br>A4<br>A5<br>A6		C ★<br>C2 ★<br>C3 ★<br>C4 ★<br>C5 ★<br>C6 ★	
Mus musculus			A<br>A2<br>A3			
Gorilla gorilla gorilla						
Homo sapiens						

The protein sequences were obtained from The National Center for Biotechnology Information (NCBI) Ensemble or UCSC Genome Browser (http://www.genome.ucsc.edu/). The domain architectures were predicted by Pfam (http://pfam.xfam.org/) program. White rectangle indicates the GSDM pore forming domain and black rectangle stands for GSDM PUB domain. Black star indicates the GSDM homologs firstly emerged in distinct evolutionary stages. GSDM, gasdermin; PJVK, pejvakin. E-L indicates the full-length GSDME. E-S, E-S1, E-S2 et al indicate the GSDME alternative splicing isoforms. Ea and Eb indicate distinct GSDME genes arrayed on the same scaffold. A to A6 or C to C6 indicate the expanded GSDMA and GSDMC genes in specific species, respectively.
