# Supplementary material for: Characterization of GSDME in amphioxus provides insights into the functional evolution of GSDM-mediated pyroptosis
Source: PLoS Biol. 2023 May 3;21(5):e3002062. doi: 10.1371/journal.pbio.3002062 (PMC10155998; doi:10.1371/journal.pbio.3002062)
Supplement: S4 Table — (PDF) [file pbio.3002062.s012.pdf]

S4 Table. Reagents, antibodies, cells and microbes used in this study

Names	Source	Catalogue number
Anti-Flag	Sigma-Aldrich	F3165
Anti-GAPDH	Proteintech	60004-1-Ig
Anti-Caspase3	Cell Signaling Technology	9662S
Anti-6 × His	Sangon Biotech	D191001
Anti-Myc	Sigma-Aldrich	M4439
Anti-Vinculin	Proteintech	66305-1-Ig
Anti-HA	Proteintech	H9658
HRP-conjugated Affinipure Goat Anti-Mouse	Proteintech	SA00001-1
HRP-conjugated Affinipure Goat Anti-Rabbit	Proteintech	SA00001-2
Recombinant human TNFα	Invivogen	rcyc-htnfa
Cycloheximide (CHX)	Sigma-Aldrich	01810
Annexin V-FITC Apoptosis Detection Kit	Beyotime	C1062
LDH Cytotoxicity Assay Kit	Beyotime	C0016
Caspase-3 (human), (recombinant) (active)	Enzo Life Sciences	ALX-201-059-U100
Human IL-6 ELISA Kit	Proteintech	KE00007
IPTG	Sangon Biotech	A100487-0001
Hoechst 33258	Beyotime	C1011
DAPI	Beyotime	C1006
Membrane Lipid Strips	Echelon Biosciences	P-6002
ANTI-FLAG ® M2 Affinity Gel	Sigma-Aldrich	A2220
HEK 293T	ATCC	
HeLa	ATCC	
Bacillus megaterium	BNCC 190686	
DH5α	Vazyme	C502-01
BL21(DE3)	Vazyme	C504-03
