# Supplementary material for: Characterization of GSDME in amphioxus provides insights into the functional evolution of GSDM-mediated pyroptosis
Source: PLoS Biol. 2023 May 3;21(5):e3002062. doi: 10.1371/journal.pbio.3002062 (PMC10155998; doi:10.1371/journal.pbio.3002062)
Supplement: S5 Table — (PDF) [file pbio.3002062.s013.pdf]

S5 Table. Primers used for RT-PCR in this study

Primers	Sequence (5’- 3’)
qBbGAPDH-F	CAAGGCTGTAGGCAAGGTCAT
qBbGAPDH-R	CTTCTTCAGTCGGCAGGTCAG
qBbGSDME-F	CAAACAAGTGTCGCCCACTG
qBbGSDME-R	CAGCTCACAGCCACGTCTAT
