# Supplementary material for: Characterization of GSDME in amphioxus provides insights into the functional evolution of GSDM-mediated pyroptosis
Source: PLoS Biol. 2023 May 3;21(5):e3002062. doi: 10.1371/journal.pbio.3002062 (PMC10155998; doi:10.1371/journal.pbio.3002062)
Supplement: S1 Appendix — (PDF) [file pbio.3002062.s014.pdf]

S1 Appendix. The sequence alignment of GSDME and PJVK with secondary structure elements. Figure is shown by ENDscript. The species abbreviations were listed in S2 table. Squiggles indicate helices, arrows stand for β-strands and TT letters stand for turns. The blue triangle indicates the SNP variants of HsGSDME, corresponding to S7F Fig.

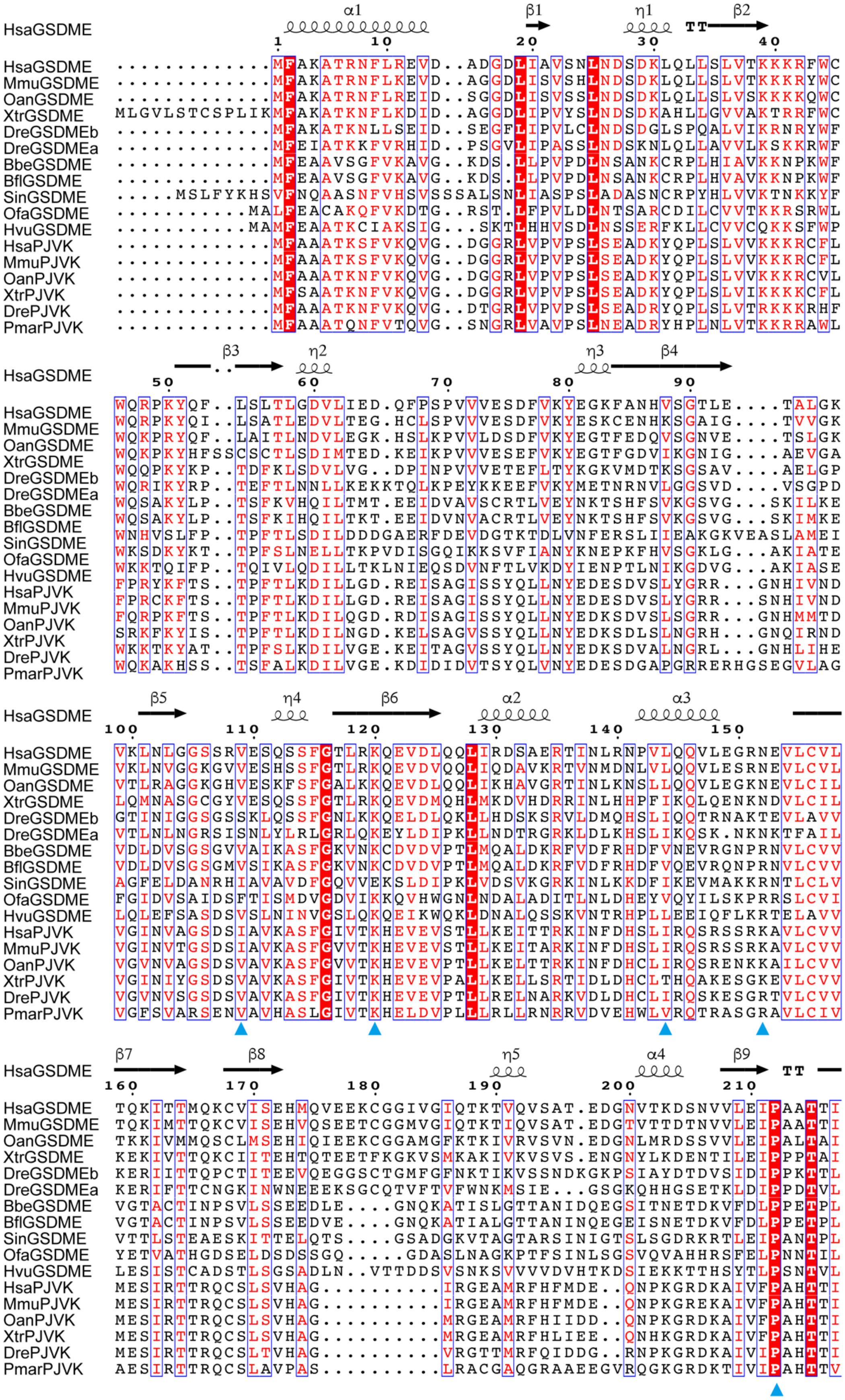
α1 β1 η1 β2 TT
1 10 20 30 40
HsaGSDME ............MFAKATRNFLREVD..ADGDLIAVSNLNDSDKLQLLSLVTKKKRFWC
MmuGSDME ............MFAKATRNFLKEVD..AGGDLISVSHLNDSDKLQLLSLVTKKKRYWC
OanGSDME ............MFAKATRNFLREID..SGGDLISVSSLNDSDKLQLLSLVSKKKKQWC
XtrGSDME MLGVLSTCSPLIKMFAKATKNFLKDID..AGGDLIPVYSLNDSDKAHLLGVVAKTRRFWC
DreGSDMEb ............MFAKATKNLLSEID..SEGFLIPVLCLNDSDGLSPQALVIKRNRYWF
DreGSDMEa ............MFEIATKKFVRHID..PSGVLIPASSLNDSKNLQLLAVVLKSKKRWF
BbeGSDME ............MFEAAVSGFVKAVG..KDS.LLPVPDLNSANKCRPLHIAVKKNPKWF
BflGSDME ............MFEAAVSGFVKAVG..KDS.LLPVPDLNSANKCRPLHVAVKKNPKWF
SinGSDME .....MSLFYKHSVFNQAASNFVHSVSSSALSNLIASPSLADASNCRPYHLVVKTNKKYF
OfaGSDME ..........MALFEACAKQFVKDTG..RST.LFPVLDLNTSARCDILCVVTKKRSRWL
HvuGSDME ..........MAMFEAATKCIAKSIG...SKTLHHVSDLNSSERFKLLCVVCQKKSFWP
HsaPJVK ............MFAATKSFVKQVG..DGGRLVPVPSLSEADKYQPLSLVVKKKRCFL
MmuPJVK ............MFAATKSFVKQVG..DGGRLVPVPSLSEADKYQPLSLVVKKKRCFL
OanPJVK ............MFAATKNFVKQVG..DGGRLVPVPSLSEADKYQPLSLVVKKKRCVL
XtrPJVK ............MFSATKNFVKQVG..DGGRLVPVPSLSEADKYQPLSLVIKKKKCFL
DrePJVK ............MFAATKNFVKQVG..DTGRLVPVPSLSEADRYQPLSLVTRKKKRHF
PmarPJVK ............MFAATQNFVTQVG..SNGRLVAVPSLNEADRYHPLNLVTKKRRAWL
HsaGSDME β3 η2 η3 β4
50 60 70 80 90
HsaGSDME WQRPKYQF..LSLTLGDVLIED.QFPSPVVVESDFVKYEGKFANHVSGTLE....TALGK
MmuGSDME WQRPKYQI..LSATLEDVLTEG.HCLSPVVVESDFVKYESKCENHKSGAIG....TVVGK
OanGSDME WQKPRYQF..LAITLNDVLEGK.HSLKPVVLDSDFVKYEGTFEDQVSGNVE....TSLGK
XtrGSDME WQKPKYHFSSCSCTLSDIMTED.KEIKPVVVESEFVKYEGTFGDVIKGNIG....AEVGA
DreGSDMEb WQQPKYKP..TDFKLSDVLVG..DPINPVVVETEFLTYKGKVMDTKSGSAV....AELGP
DreGSDMEa WQRIKYRP..TEFTLNNLLKEKKTQLKPEYKKEEFVKYMETNRNVLGGSVD....VSGPD
BbeGSDME WQSAKYLP..TSFKVHQILTMT.EEIDVAVSCRTLVEYNKTSHFSVKGSVG...SKILKE
BflGSDME WQSAKYLP..TSFKIHQILTKT.EEIDVNVACRTLVEYNKTSHFSVKGSVG...SKIMKE
SinGSDME WNHVSLFP..TPFTLSDILDDDGAERFDEVDGTKTDLVNFERSLIIEAKGKVEASLAMEI
OfaGSDME WKSDKYKT..TPFSLNELLTKPVDISGQIKKSVFIANYKNEPKFHVSGKLG...AKIATE
HvuGSDME WKKTQIFP..TQIVLQDILLTKLNIEQSDVNFTLVKDYIENPTLNIKGDVG...AKIASE
HsaPJVK FPRYKFTS..TPFTLKDILLGD.REISAGISSYQLLNYEDESDVSLYGRR...GNHIVND
MmuPJVK FPRCKFTS..TPFTLKDILLGD.REISAGISSYQLLNYEDESDVSLYGRR...SNHIVND
OanPJVK FQRPKFTS..TPFTLKDILQGD.RDISAGISSYQLLNYEDKSDVSLSGRR...GNHMMTD
XtrPJVK SRKFKYIS..TPFTLKDILNGD.KELSAGVSSYQLLNYEDKSDLSLNGRH...GNQIRND
DrePJVK WKKTKYAT..TPFSLKDILVGE.KEITAGVSSYQLLNYEDKSDVALNGRL...GNHLIHE
PmarPJVK WQKAKHSS..TSFALKDILVGE.KDIDIDVTSYQLVNYEDESDGAPGRRERHGSEGVLAG
HsaGSDME β5 η4 β6 α2 α3
100 110 120 130 140 150
HsaGSDME VKLNLGGSSRVESQSSFGTLRKQEVDLQQLIRDSAERTINLRNPVLQQVLEGRNEVLCVL
MmuGSDME VKLNVGGKGVVESHSSFGTLRKQEVDVQQLIQDAVKRTVNMDNLVLQQVLESRNEVLCVL
OanGSDME VTLRAGGKGHVESKFSFGALKKQEVDLQQLIKHAVGRTINLKNSLLQQVLEGKNEVLCIL
XtrGSDME LQMNASGCGYVESQSSFGTLRKQEVDMQHLMKDVHDRRINLHHPFIKQLQENKNDVLCIL
DreGSDMEb GTINIGGSGSSKLQSSFGNLKKQELDLQKLLHDSKSRVLDMQHSLIQQTRNAKTEVLAVV
DreGSDMEa VTLNLNGRSISNLYLRLGRLQKEYLDIPKLLNDTRGRKLDLKHSLIKQSK.NKNKTFAIL
BbeGSDME VDLDVSGSGVVAIKASFGKVNKCDVDVPTLMQALDKRFVDFRHDFVNEVRGNPRNVLCVV
BflGSDME VDLDVSGSGMVSIKASFGKVNKCDVDVPTLMQALDKRFVDFRHDFVQEVRQNPRNVLCVV
SinGSDME AGFELDANRHIAVAVDFGQVVEKSLDIPKLVDSVKGRKINLKKDFIKEVMAKKRNTLCLV
OfaGSDME FGIDVSAIDSFTISMDVGDVIKKQVHWGNLNDALADITLNLDHEYVQYILSKPRRSLCVI
HvuGSDME LQLEFSASDSVSLNINVGSLQKQEIKWQKLDNALQSSKVNTRHPLLEEIQFLKRTELAVV
HsaPJVK VGINVAGSDSIAVKASFGIVTKHEVEVSTLLKEITTRKINFDHSLIRQSRSSRKAVLCVV
MmuPJVK VGINVTGSDSIAVKASFGVVTKHEVEVSTLLKEITARKINFDHSLIRQSRSSRKAVLCVV
OanPJVK VGVNVAGSDSVAVKASFGVVTKHEVEVPTLLKELTTRKINFDHCLIRQSRENKKAILCVV
XtrPJVK VGINIYGSDSVAVKASFGIVTKHEVEVPALLKELLSRTIDLDHCLTHQAKESGKEVLCVV
DrePJVK VGVNVSGSDSVAVKASFGIVTKHEVEVPTLLRELNARKVDLDHCLIRQSKESGRTVLCVV
PmarPJVK VGFSVARSENVAVHASLGIVTKHELDVPLLLRLLRNRRVDVEHWLVRQTRASGRAVLCIV
HsaGSDME β7 β8 η5 α4 β9 TT
160 170 180 190 200 210
HsaGSDME TQKITTMQKCVISEHMQVEEKCGGIVGIQTKTVQVSAT.EDGNVTKDSNVVLEIPAATTI
MmuGSDME TQKIMTTQKCVISEHVQSEETCGGMVGIQTKTIQVSAT.EDGTVTTDTNVVLEIPAATTI
OanGSDME TKKIVMMQSCLMSEHIQIEEKCGGAMGFKTKIVRVSVN.EDGNLMRDSSVVLEIPALTAI
XtrGSDME KEKIVTTQKCIITEHTQTEETFKGKVSMKAKIVKVSVS.ENGNYLKDENTILEIPPPTAI
DreGSDMEb KERIITTQPCTITEEVQEGGSCTGMFGFNKTIKVSSNDKGKPSIAYDTDVSIDIPPKTTL
DreGSDMEa KERIFTTCNGKINWNEEEKSGCQTVFTVFWNKMSIE...GSGKQHHGSETKLDIPPDTVL
BbeGSDME VGTACTINPSVLSSEEDLE....GNQKATISLGTTANIDQEGSITNETDKVFDLPPETPL
BflGSDME VGTACTINPSVLSSEEDVE....GNQKATIALGTTANINQEGEISNETDKVFDLPPETPL
SinGSDME VTTLSTEAESKITTELQTS....GSADGKVTAGTARSINIGTSLSGDRKRTLEIPANTPL
OfaGSDME YETVATHGDSELDSDSSGQ.....GDASLNAGKPTFSINLSGSVQVAHHRSFELPNNTIL
HvuGSDME LESISTCADSTLSGSADLN..VTTDDSVSNKSVVVVDVHTKDSIEKKTTHSYTLPSNTVL
HsaPJVK MESIRTTRQCSLSVHAG..........IRGEAMRFHFMDE..QNPKGRDKAIVFPAHTTI
MmuPJVK MESIRTTRQCSLSVHAG..........IRGEAMRFHFMDE..QNPKGREKAIVFPAHTTI
OanPJVK MESIRTTRQCSLSVHAG..........MRGEAMRFHIIDD..QNPKGRDKAIVFPAHTTI
XtrPJVK MESIRTTRQCSLSVHAG..........MRGEAMRFHLIEE..QNHKGRDKAIVFPAHTTI
DrePJVK MESIRTTRQCSLTVHAG..........VRGTTMRFQIDDG..RNPKGRDKAIVIPAHTTI
PmarPJVK AESIRTTRQCSLAVPAS..........LRACGAQGRAAEEGVRQGKGRDKTIVIPAHTTV

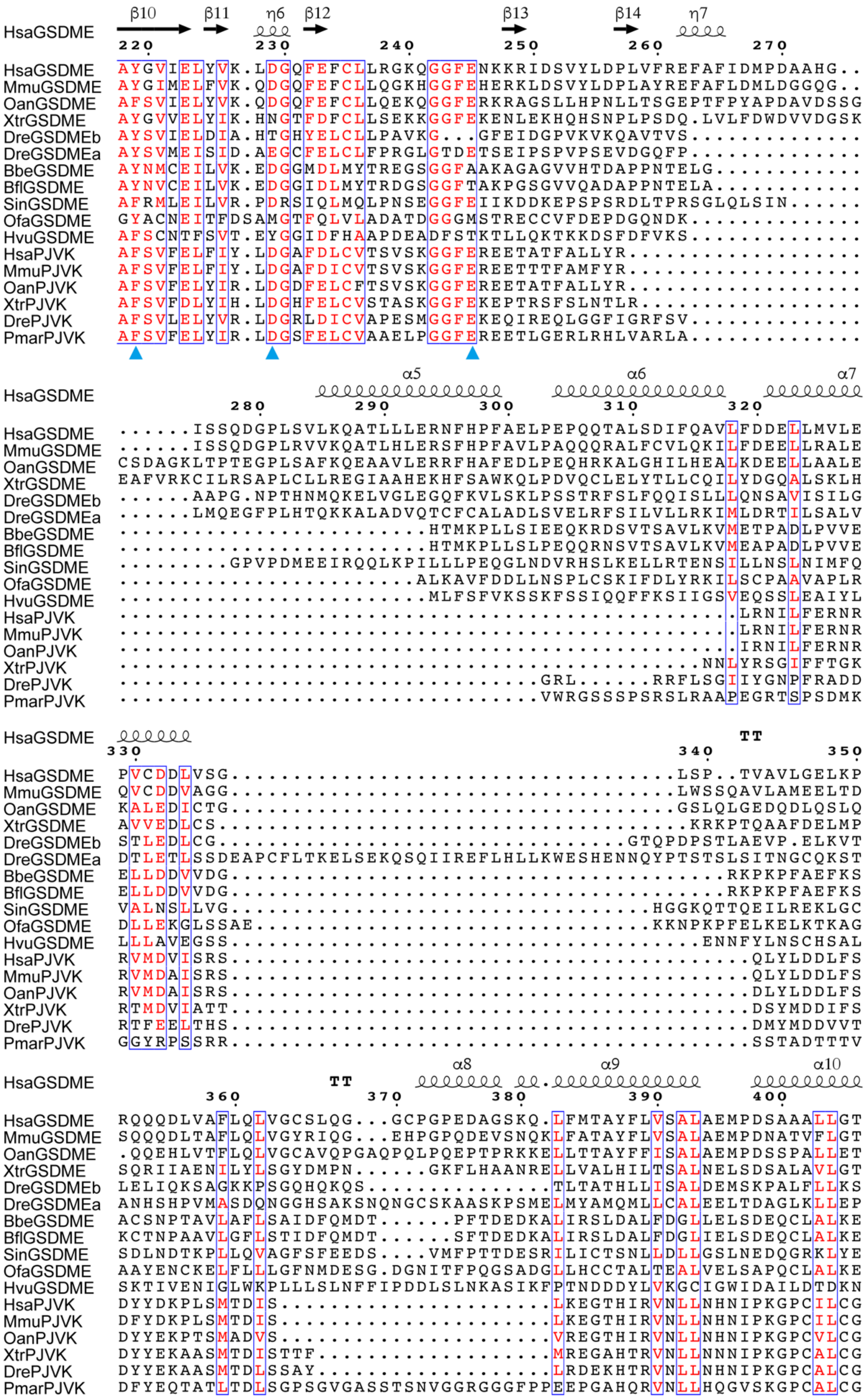
HsaGSDME β10 β11 η6 β12 β13 β14 η7
220 230 240 250 260 270
HsaGSDME AYGVIELYVK.LDGQFEFCLLRGKQGGFENKKRIDSVYLDPLVFREFAFIDMPDAAHG..
MmuGSDME AYGIMELFVK.QDGQFEFCLLQGKHGGFEHERKLDSVYLDPLAYREFAFLDMLDGGQG..
OanGSDME AFSVIELYVK.QDGQFEFCLLQEKQGGFERKRAGSLLHPNLLTSGEPTFPYAPDAVDSSG
XtrGSDME AYGVVELYIK.HNGTFDFCLLSEKKGGFEKENLEKHQHSNPLPSDQ.LVLFDWDVVDGSK
DreGSDMEb AYSVIELDIA.HTGHYELCLLPAVKG...GFEIDGPVKVKQAVTVS..............
DreGSDMEa AYSVMEISID.AEGCFELCLFPRGLGTDETSEIPSPVPSEVDGQFP..............
BbeGSDME AYNMCEILVK.EDGGMDLMYTREGSGGFAAKAGAGVVHTDAPPNTELG............
BflGSDME AYNVCEILVK.EDGGIDLMYTRDGSGGFTAKPGSGVVQADAPPNTELA............
SinGSDME AFRMLEILVR.PDRSIQLMQLPNSEGGFEIIKDDKEPSPSRDLTPRSGLQLSIN......
OfaGSDME GYACNEITFDSAMGTFQLVLADATDGGGMSTRECCVFDEPDGQNDK..............
HvuGSDME AFSCNTFSVT.EYGGIDFHAAPDEADFSTKTLLQKTKKDSFDFVKS..............
HsaPJVK AFSVFELFIY.LDGAFDLCVTSVSKGGFEREETATFALLYR...................
MmuPJVK AFSVFELFIY.LDGAFDICVTSVSKGGFEREETTTFAMFYR...................
OanPJVK AFSVFELYIR.LDGDFELCFTSVSKGGFEREETATFALLYR...................
XtrPJVK AFSVFDLYIH.LDGHFELCVSTASKGGFEKEPTRSFSLNTLR..................
DrePJVK AFSVLELYVR.LDGRLDICVAPESMGGFEKEQIREQLGGFIGRFSV..............
PmarPJVK AFSVFELYIR.LDGSFELCVAAELPGGFEREETLGERLRHLVARLA..............
HsaGSDME α5 α6 α7
280 290 300 310 320
HsaGSDME ......ISSQDGPLSVLKQATLLLERNFHPFAELPEPQQTALSDIFQAVLFDDELLMVLE
MmuGSDME ......ISSQDGPLRVVKQATLHLERSFHPFAVLPAQQQRALFCVLQKILFDEELLRALE
OanGSDME CSDAGKLTPTEGPLSAFKQEAAVLERRFHAFEDLPEQHRKALGHILHEALKDEELLAALE
XtrGSDME EAFVRKCILRSAPLCLLREGIAAHEKHFSAWKQLPDVQCLELYTLLCQILYDGQQALSKLH
DreGSDMEb ......AAPG.NPTHNMQKELVGLEGQFKVLSKLPSSTRFSLFQQISLLLQNSAVISILG
DreGSDMEa ......LMQEGFPLHTQKKALADVQTCFCALADLSVELRFSILVLLRKIMLDRTILSALV
BbeGSDME .......................HTMKPLLSIEEQKRDSVTSAVLKVMETPADLPVVE
BflGSDME .......................HTMKPLLSLPEQQRNSVTSAVLKVMEAPADLPVVE
SinGSDME .........GPVPDMEEIRQQLKPILLLPEQGLNDVRHSLKELLRTENSILLNSLNIMFQ
OfaGSDME .....................ALKAVFDDLLNSPLCSKIFDLYRKILSCPAAVAPLR
HvuGSDME .......................MLFSFVKSSKFSSIQQFFKSIIGSVEQSSLEAIYL
HsaPJVK ....LRNILFERNR
MmuPJVK ....LRNILFERNR
OanPJVK ....IRNILFERNR
XtrPJVK ....NNLYRSGIFFTGK
DrePJVK ....GRL......RRFLSGIIYGNPFRADD
PmarPJVK ..................................VWRGSSSPSRSLRAAPEGRTSPSDMK
HsaGSDME TT
330 340 350
HsaGSDME PVCDDLVSG..............................LSP..TVAVLGELKP
MmuGSDME QVCDDVAGG..............................LWSSQAVLAMEELTD
OanGSDME KALEDICTG..............................GSLQLGEDQDLQSLQ
XtrGSDME AVVEDLCS.................................KRKPTQAAFDELMP
DreGSDMEb STLEDLCG............................GTQPDPSTLAEVP.ELKVT
DreGSDMEa DTLETLSSDEAPCFLTKELSEKQSQIIREFLHLLKWESHENNQYPTSTSLSITNGCQKST
BbeGSDME ELLDDVVDG..................................RKPKPFAEFKS
BflGSDME ELLDDVVDG..................................RKPKPFAEFKS
SinGSDME VALNSLLVG............................HGGKQTTQEILREKLGC
OfaGSDME DLLEKGLSSAE..........................KKNPKPFELKELKTKAG
HvuGSDME LLLAVEGSS..................................ENNFYLNSCHSAL
HsaPJVK RVMDVISRS.....................................QLYLDDLFS
MmuPJVK RVMDAISRS.....................................QLYLDDLFS
OanPJVK RVMDAISRS.....................................DLYLDDLFS
XtrPJVK RTMDVIATT.....................................DSYMDDIFS
DrePJVK RTFEELTHS.....................................DMYMDDVVT
PmarPJVK GGYRPSSRR.....................................SSTADTTTV
HsaGSDME TT α8 α9 α10
360 370 380 390 400
HsaGSDME RQQQDLVAFLQLVGCSLQG...GCPGPEDAGSKQ.LFMTAYFLVSALAEMPDSAAALLGT
MmuGSDME SQQQDLTAFLQLVGYRIQG...EHPGPQDEVSNQKLFATAYFLVSALAEMPDNATVFLGT
OanGSDME .QQEHLVTFLQLVGCAVQPGAQPQLPQEPTPRKKELLTTAYFFISALAEMPDSSPALLET
XtrGSDME SQRIIAENILYLSGYDMPN......GKFLHAANRELLVALHILTSALNELSDSALAVLGT
DreGSDMEb LELIQKSAGKKPSGQHQKQS.........TLTATHLLISALDEMSKPALFLLKS
DreGSDMEa ANHSHPVMASDQNGGHSAKSNQNGCSKAASKPSMELMYAMQMLLCALEELTDAGLKLLEP
BbeGSDME ACSNPTAVLAFLSAIDFQMDT......PFTDEDKALIRSLDALFDGLLELSDEQCLALKE
BflGSDME KCTNPAAVLGFLSTIDFQMDT......SFTDEDKALIRSLDALFDGLIELSDEQCLALKE
SinGSDME SDLNDTKPLLQVAGFSFEEDS....VMFPTTDESRILICTSNLLDLLGSLNEDQGRKLYE
OfaGSDME AAYENCKELFLLLGFNMDESG.DGNITFPQGSADGLLHCCTALTEALVELSAPQCLALKE
HvuGSDME SKTIVENIGLWKPLLLSLNFFIPDDLSLNKASIKFPTNDDDYLVKGCIGWIDAILDTDKN
HsaPJVK DYYDKPLSMTDIS....................LKEGTHIRVNLLNHNIPKGPCILCG
MmuPJVK DFYDKPLSMTDIS....................LKEGTHIRVNLLNHNIPKGPCILCG
OanPJVK DYYEKPTSMADVS....................VREGTHIRVNLLNHNIPKGPCVLCG
XtrPJVK DYYEKAASMTDISTTF.................MREGAHTRVNLLNNNIPKGPCALCG
DrePJVK DYYEKAASMTDLSSAY.................LRDEKHTRVNLLNHNIPKGPCALCG
PmarPJVK DFYEQTATLTDLSGPSGVGASSTSNVGGRGGGFPPEEPGAHQRVNLLHQGVSKGPCALCG

```
                 α11              β15   α12   η8        α13             β16     β17
HsaGSDME
             410          420         430         440         450         460
HsaGSDME    CCKLQIIPTLCHLLRALSDDGVSDLEDPTLTPLKDTERFGIVQRLFASADISLERLKSSV
MmuGSDME    CCKLHVISSLCCLLHALSDDSVCDFHNPTLAPLRDTERFGIVQRLFASADIALERMQFSA
OanGSDME    CRELQLVPALCRLPRITAADGSSALQDPTLAPLADLERFGIVQRLFALSNINLERTKSSV
XtrGSDME    CCELQLLPVFSALMNMSSDEGLCSTTEPALMDFLDQERFYVSQKLFALFNIELEIKEDFI
DreGSDMEb   CCSYTTLQALLHLVQNMALNEKSSLKDAALDVLADEVVFKNITSLFNSCNVMLLKDDNSL
DreGSDMEa   FCTSEHLSSLQDLVIHLTSIMP.LCKNTVPVSLQNEDEFHRKEELFKSCNVLLKKENDSL
BbeGSDME    CNSNFAPAILHVLEQGIAGHPVPLDDPAISILYVKSNNPGQDFLEALDFGIQDVGNKKSL
BflGSDME    CNSNFAPAILHVLEQGIAGNPVPLDDPAISILYVKSNNPGQDFIEAMDFGIQDVGNKKAL
SinGSDME    CSEEEKRSITKVIAEALAVERLPLDQPSVSDLFNGSRRAESLVFSLGFQLERTDGHWYLS
OfaGSDME    VTSEYLEPLLYLLKNVMYSKDTSVDDPLLQRVWTHSANPAKNLLLSLGFDQVITEGNNKL
HvuGSDME    LRHYLVQFSDEHLKLLLHIVESKILNKEINEEIQINLMFSENQSTKLFLNGIGFVFETEE
HsaPJVK     MGNFKRETVYGCFQCSVDGQKYVRLHAVPCFDIWHKRMK.....................
MmuPJVK     MGNLKRETVYGCFQCSVDGVKYVRLHAVPCFDIWHKRMK.....................
OanPJVK     MGNHKRETVYGCFDCSIDGQKYVRLHVVPCFDLWHKRMR.....................
XtrPJVK     MGNSKRETVYGCFECSFNGQKYVRLHAVPCFDLWHKRMK.....................
DrePJVK     MGHQRRETVYGCLECTSGGNKYVRLHAVPC..............................
PmarPJVK    LGARPRDTVYGCVECAWGGHKYVRLHAVPCFDLWHRKMKS....................

                η9        α14
HsaGSDME
             470          480         490
HsaGSDME    KAVILKDSKVFPLLLCITLNGLCALGREHS.............
MmuGSDME    KATILKDSCIFPLILHITLSGLSTLSKEHEEELCQSGHATGQD
OanGSDME    KATIVKEPKFRPLILYIVLSGLCALDRHRQNQPQ.........
XtrGSDME    YAATAEDPGFLPLILFIVITGLQLLKRD...............
DreGSDMEb   ITKISNPEDRLPLMLCIAVKGLASLAPHV..............
DreGSDMEa   TSEVTCSVGFLPLVLCIAIHGLASLSAV...............
BbeGSDME    VTEDDGKRLYAAQQAVYGLWGGL....................
BflGSDME    VTEDDGKTLYAAQQAVYGLWGGL....................
SinGSDME    YSTSEMVEDDLNPAGLLVIMCALWDQK................
OfaGSDME    LQLTWDSEHISLEDTYYVAVFVLCSE.................
HvuGSDME    EKEVMTYPSEDHQKLLDAFINIYLLSA................
HsaPJVK     ...........................................
MmuPJVK     ...........................................
OanPJVK     ...........................................
XtrPJVK     ...........................................
DrePJVK     ...........................................
PmarPJVK    ...........................................
```
