# Supplementary material for: Characterization of GSDME in amphioxus provides insights into the functional evolution of GSDM-mediated pyroptosis
Source: PLoS Biol. 2023 May 3;21(5):e3002062. doi: 10.1371/journal.pbio.3002062 (PMC10155998; doi:10.1371/journal.pbio.3002062)
Supplement: S2 Appendix — (PDF) [file pbio.3002062.s015.pdf]

Supplementary materials and methods

Animals, reagents, antibodies and microbes. Adult Chinese amphioxus (~1 year old) *Branchiostoma belcheri* were captured by using dense nets from the sea area nearby Zhanjiang city, China. After capturing, lancelets were put into a sea water-containing tank and transported to the laboratory. Lancelets were cultured in a laboratory incubator under modeled wild conditions. All the experimental protocols for handling of adult Chinese amphioxus were approved by the Institutional Animal Care and Use Committee of Sun Yat-sen University, Guangzhou, China. All relevant ethical rules regarding the animals were compliant in this study. For *in vivo* infection experiments, indicated numbers of healthy amphioxus adults were randomly selected and grouped without gender distinction. Reagents, antibodies, cells and microbes used in this study were listed in S4 Table.

Phylogenetic, exon-intron structure and collinearity analyses. The protein sequences were downloaded from the NCBI website (https://www.ncbi.nlm.nih.gov/) and the Ensembl database. MAFFT v7.487 was used for multiple sequence alignment and alignment trimming [1, 2]. A maximum likelihood (ML) phylogenetic tree was constructed by IQ-TREE v2.1.4 with 1000 step bootstraps [3]. The derived phylogenetic trees were visualized using Evolview v3 [4]. The exon-intron structure was described using the published method [5]. The collinearity analysis was conducted using MCScanX as described previously and graphically drawn by TBtools [6, 7]. The sequence alignment of GSDME and PJVK with secondary structure elements was shown by ENDscript [8].

Genes cloning and plasmid construction. GSDME ortholog was identified in the *Branchiostoma belcheri* (Bb) genome. Based on these sequences, BbGSDME was cloned from amphioxus *Branchiostoma belcheri* intestinal cDNAs using specific primers. The cloned BbGSDME cDNA was deposited to the GenBank database under accession numbers of OL405706. For the expression of BbGSDME, in mammalian cells, the protein coding sequence (CDS) of BbGSDME was subcloned into pEZM12). For the study of subcellular localization, the GSDM CDSs were subcloned to pEZM98 (GeneCopoeia) expression vector. For recombinant protein expressions, the CDSs of BbGSDME and its N-termini were subcloned into the His-tagged pEZB01 (GeneCopoeia) expression vector. The mutations of BbGSDME were generated using the QuickChange Lightning Multi Site-Directed Mutagenesis Kit (Agilent) according to the manufacturer's instructions. The altered regions were confirmed by Sanger sequencing.

Purification of the Flag-tagged GSDME homologs and their N-termini from 293T cells. HEK 293T cells were transfected with BbGSDME-pEZM12 plasmids and then collected in 48 h. Cells were washed by PBS and dounce-homogenized in a cell lysis buffer (20 mM Tris (pH 7.5), 150 mM NaCl, 1% TritonX-100 (v/v) and complete protease inhibitor mixture (Roche)). Lysates were cleared by centrifugation and immunoprecipitated overnight with anti-Flag M2 Affinity Gel (Sigma-Aldrich). Immunoprecipitations were washed five times with cell lysis buffer and two times with buffer A (20 mM Tris (pH 7.5), 150 mM NaCl). Samples were eluted with 5 μg/μl 3×Flag peptide (Sigma-Aldrich) according to manufacturer's instructions. The purified protein concentration was quantified using BCA Protein Assay Kit (Thermo Fisher Scientific).

***In vitro* CASPs cleavage assays.** In brief, indicated GSDMs (5 μg) was incubated with 1 U of rHsCASP 3 (Enzo Life Sciences) in 25 μl reaction buffer (50 mM HEPES (pH 7.5), 150 mM NaCl, 3 mM EDTA, 10 mM DTT and 0.005% (v/v) Tween-20). The reaction was carried out at 37°C for 1-2 hrs. Subsequently, 5× SDS loading buffer was added to the reaction mixture and heated to 100°C for 10 min. The cleaved fragments were separated by SDS-PAGE, followed by Coomassie brilliant blue staining or Western blotting.

Co-IP assays. HEK 293T cells in 6-well dishes were transfected with 4 μg DNA plasmids (2 μg/each expression vector). At 24 h post-transfection, the whole cell extracts were prepared in IP lysis buffer (50 mM Tris, pH7.4, 150 mM NaCl, 1% Nonidet P-40, 0.5% deoxycholic acid sodium salt and cocktail protease inhibitor (Roche), followed by incubating with anti-Flag beads (Sigma-Aldrich) at 4ºC for 2 hours. Then washed three times with IP lysis buffer and boiled 10 mins in 100ºC with SDS loading buffer. Analysis was conducted using SDS-PAGE followed by western blot.

Western blotting analysis. In brief, cell lysates were subjected to SDS-PAGE before being transferred to nitrocellulose (NC) membranes (Millipore) by electroblotting. The NC membranes were then blocked with PBST 5% fat-free dried milk at RT for 2 h, then probed with antibodies against Flag (Sigma-Aldrich), HA (Sigma-Aldrich) and 6×His (Sangon Biotech) at 4°C overnight. HRP-conjugated anti-rabbit and anti-mouse secondary antibodies (Proteintech) were used to probe the positive bands, which were visualized by ECL assay.

Purification of recombinant proteins from BL21 (DE3). Recombinant proteins of BbGSDME were purified from BL21 (DE3) *E. coli* strain (Vazyme). For BbGSDME protein, BL21 (DE3) cells were first transfected with BbGSDME-pEZB01 and then cultured in LB medium at 37°C with shaking at 220 rpm until the OD600 reached 0.6. After induction with IPTG (final concentration of 0.4 mM), BbGSDME was expressed as inclusion bodies in cells. Thus, BL21 (DE3) cells were harvested and lysed by ultrasonication on ice in lysis buffer (10% SDS, 50 mM Tris, 5 mM EDTA, 150 mM NaCl). The precipitates were collected and washed four times with wash buffer (50 mM Tris, 3 mM EDTA, 5% Glycerol, 1% TritonX-100). Sufficient 8 M urea was added to dissolve the precipitate at room temperature, and undissolved precipitate was removed by centrifugation. The urea was diluted to 1 M by adding sterile water to allow protein refolding. Proteins of BbGSDME were then purified using the His-tag Protein Purification Kit (Beyotime) according to the manufacturer's instructions. The purified protein concentration was quantified using BCA Protein Assay Kit (Thermo Fisher Scientific).

Rabbit antiserum preparation. Rabbit polyclonal antibody against BbGSDME was prepared by Genscript Company (JiangSu, China). First, a total of 5-6 ml blood was collected from the ear artery of healthy rabbits as pre-immune serum. The purified BbGSDME proteins diluted with normal saline and mixed with immune adjuvant by 1:1, were injected subcutaneously into the thighs and shoulders of rabbits on day 1, day 14 and day 21. On day 28, blood containing antibody was collected from carotid arteries of rabbits, stored at 37°C for 2 h and precipitated at 4°C overnight. The next day, the serum containing antibody was collected by centrifugation at 10,000 rpm for 10 min. Finally, the anti-BbGSDME immunoglobulin G (IgG) was purified by protein-A

affinity resin according to the manufacturer's instructions and its titer was detected by ELISA.

Whole-mount *in situ* hybridization. Amphioxus embryos were fixation in 4% PFA-MOPS (Sigma-Aldrich). The probe targeting *Bbgsdme* was made using Sp6 or T7 RNA polymerase (Promega) and labelled with Digoxigenin (DIG). Embryos were hybridized with target probes and detected with anti-DIG-POD (Roche). The signal was then shown with the ECL system (PerkinElmer). After staining, embryos were washed with PBS, mounted in 50% glycerol, and photographed using an inverted microscope (Olympus).

RT-PCR analysis. Total RNA of tissues, including gill, intestines, hepatic caecum, muscle and skin from amphioxus were isolated using TRIzol (Invitrogen) or RNeasy Kit (Qiagen) according to the manufacturer's instructions, and reverse transcribed into cDNA using Prime Script RT reagent Kit with gDNA Eraser (TaKaRa) and random primers (TaKaRa). RT-PCR assays were performed using primers listed in S5 Table. The abundance of gene mRNA normalized against GAPDH.

***In vitro* bacterial killing assay**. *Edwardsiella anguillarum* (EIB202) was cultured overnight in TSB and diluted in the next day at 1:100 in TSB until OD_{600} reached 1.0. Then 5 ml of the culture was centrifuged at 5000 g for 10 min, washed once in PBS and resuspended in PBS at 1×10^9 CFU/ml. The expression plasmids of HsGSDMB, BbGSDME and their N-termini were transfected into 293T cells seeded on six-well plates. After 24 h transfection, the cells were collected and lysed by ultrasonication in

PBS. Then the supernatant was centrifugated at 12,000 g for 10 min and filtered by a 0.22 μm filter. For the bacterial killing assay, EIB202 (1×10^6 in 3 μl) was incubated with 5 μl cell lysates containing indicated protein in 25 μl reactions which were set up in PBS at 37 °C for 2 h. The reactions were serially diluted from 10^1 to 10^6, and 5 μl serial dilutions were plated on TSB agar to determine recovered CFU.

SI References

1. Katoh K, Misawa K, Kuma K, Miyata T. MAFFT: a novel method for rapid multiple sequence alignment based on fast Fourier transform. Nucleic Acids Res. 2002;30(14):3059-3066.

2. Capella-Gutierrez S, Silla-Martinez JM, Gabaldon T. trimAl: a tool for automated alignment trimming in large-scale phylogenetic analyses. Bioinformatics. 2009;25(15):1972-1973.

3. Trifinopoulos J, Nguyen LT, von Haeseler A, Minh BQ. W-IQ-TREE: a fast online phylogenetic tool for maximum likelihood analysis. Nucleic Acids Res. 2016;44(W1):W232-235.

4. Subramanian B, Gao S, Lercher MJ, Hu S, Chen WH. Evolview v3: a webserver for visualization, annotation, and management of phylogenetic trees. Nucleic Acids Res. 2019;47(W1):W270-W275.

5. Sanchez D, Ganfornina MD, Gutierrez G, Marin A. Exon-intron structure and evolution of the Lipocalin gene family. Mol Biol Evol. 2003;20(5):775-783.

6. Wang Y, Li J, Paterson AH. MCScanX-transposed: detecting transposed gene duplications based on multiple colinearity scans. Bioinformatics. 2013;29(11):1458-1460.

7. Chen C, Chen H, Zhang Y, Thomas HR, Frank MH, He Y, et al. TBtools: An Integrative Toolkit Developed for Interactive Analyses of Big Biological Data. Mol Plant. 2020;13(8):1194-1202.

8. Gouet P, Robert X, Courcelle E. ESPript/ENDscript: Extracting and rendering sequence and 3D information from atomic structures of proteins. Nucleic Acids Res. 2003;31(13):3320-3323.
