# Supplementary material for: Characterization of GSDME in amphioxus provides insights into the functional evolution of GSDM-mediated pyroptosis
Source: PLoS Biol. 2023 May 3;21(5):e3002062. doi: 10.1371/journal.pbio.3002062 (PMC10155998; doi:10.1371/journal.pbio.3002062)

Fig 2

Fig 2E

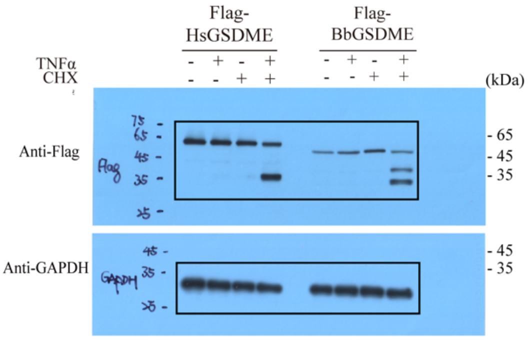

Fig 2F

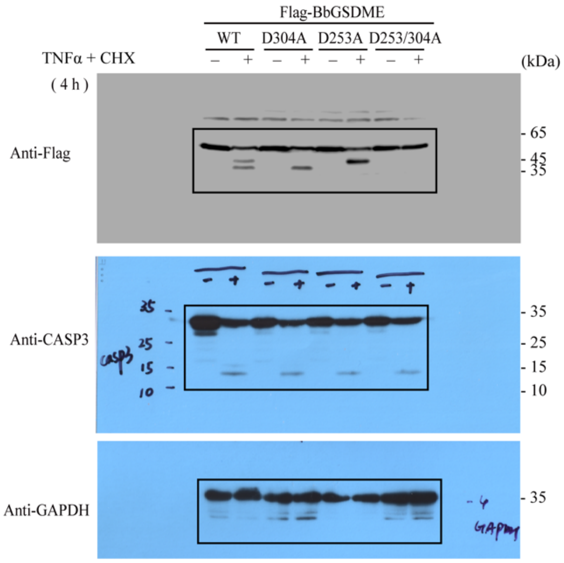

Fig 2G

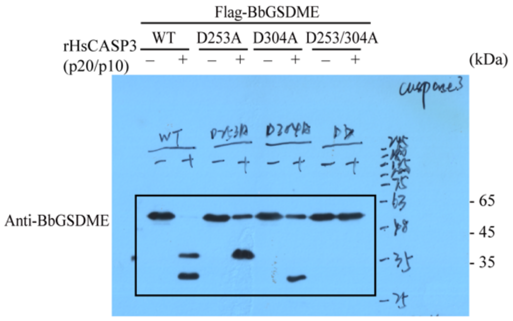

Fig 2J

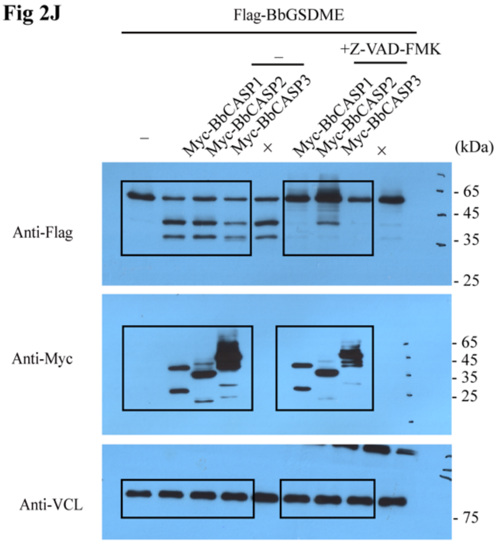

Fig 2K

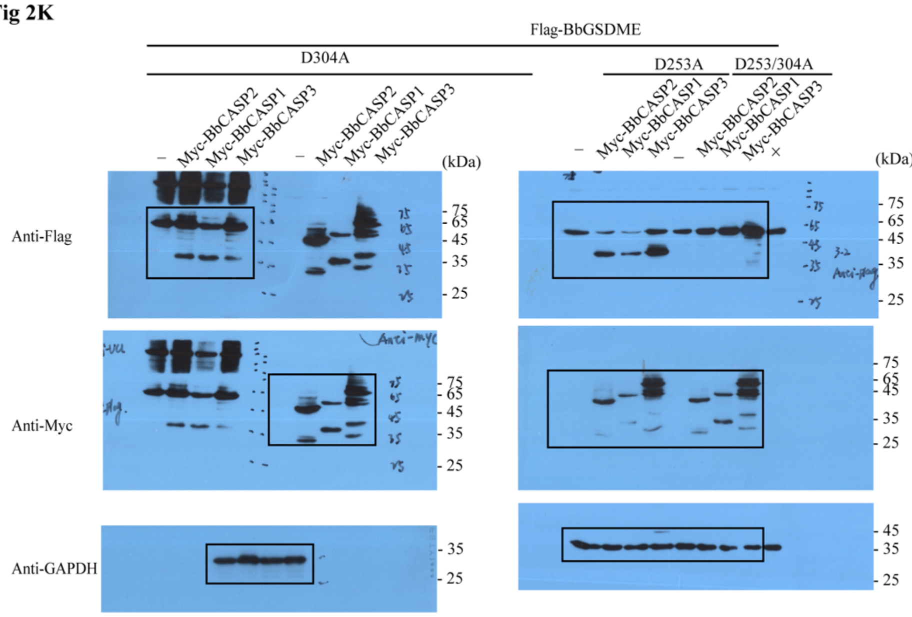

Fig 2M

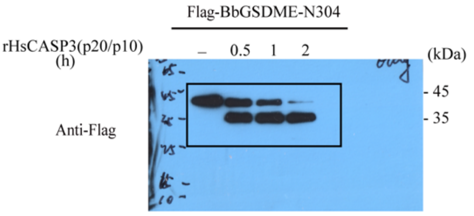

Fig 2N

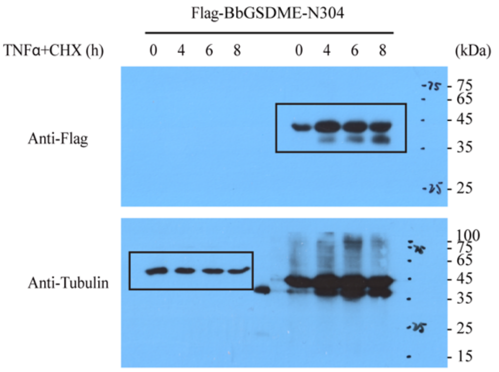

Fig 2O

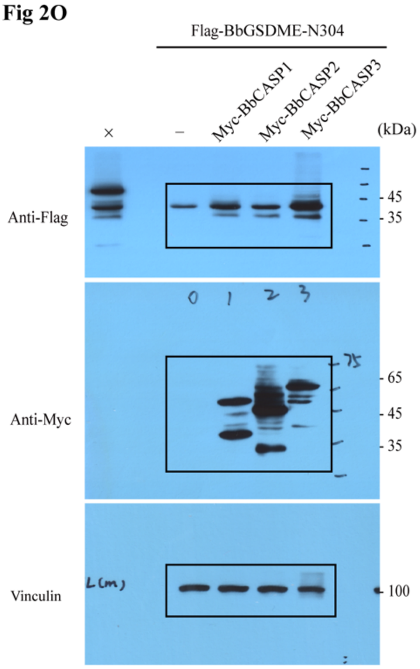

Fig 3

Fig 3E

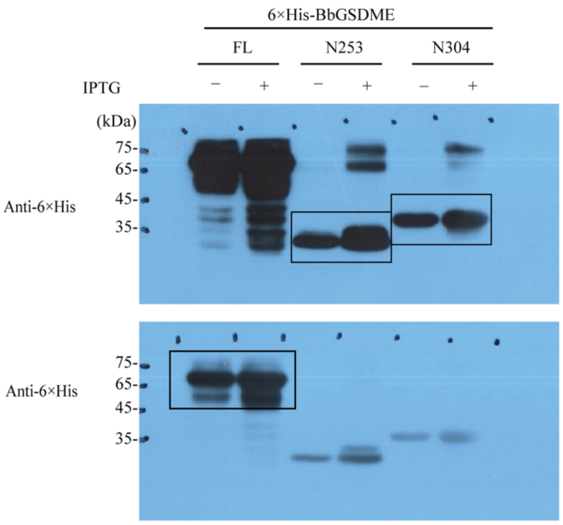

Fig 3H

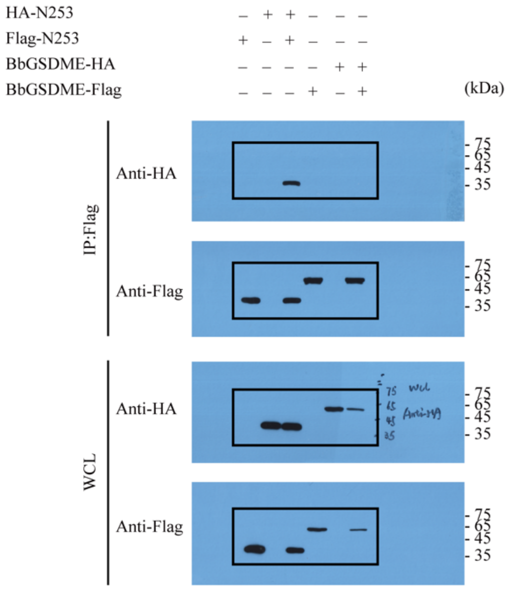

Fig 3I

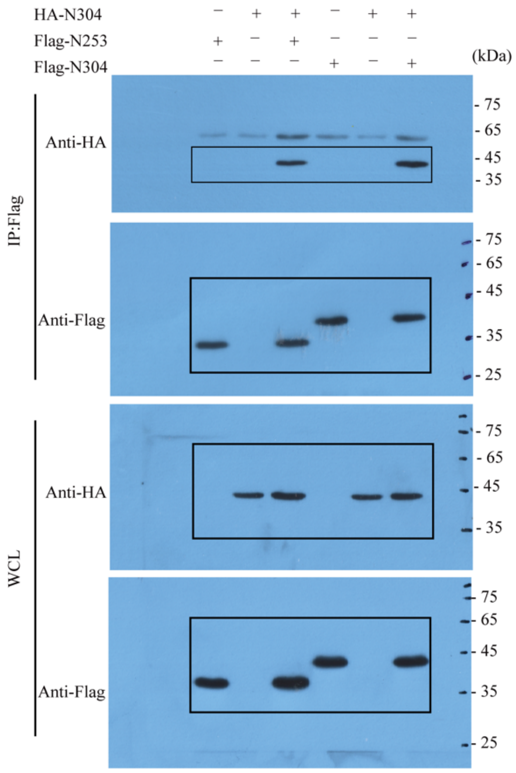

Fig 4

Fig 4A

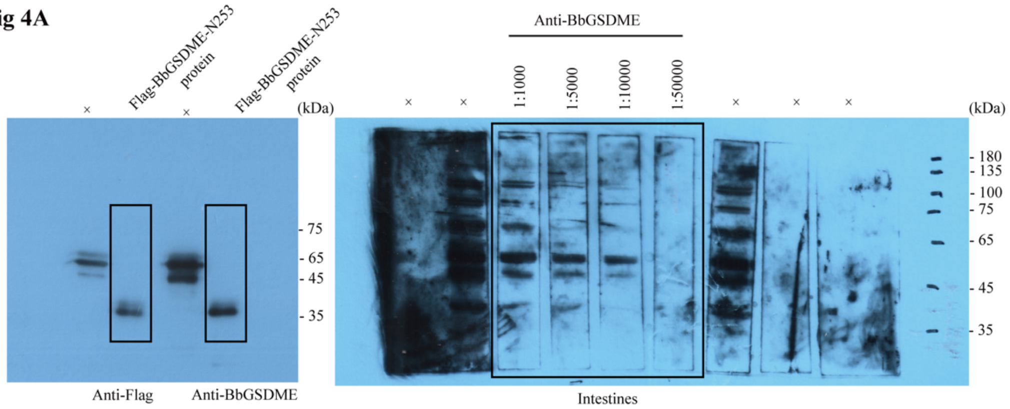

Fig 4B

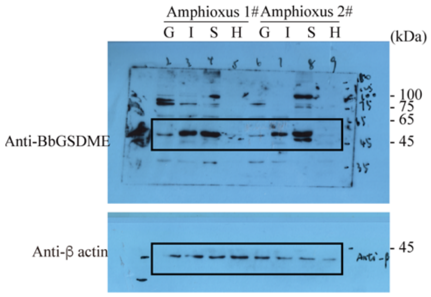

Fig 4C

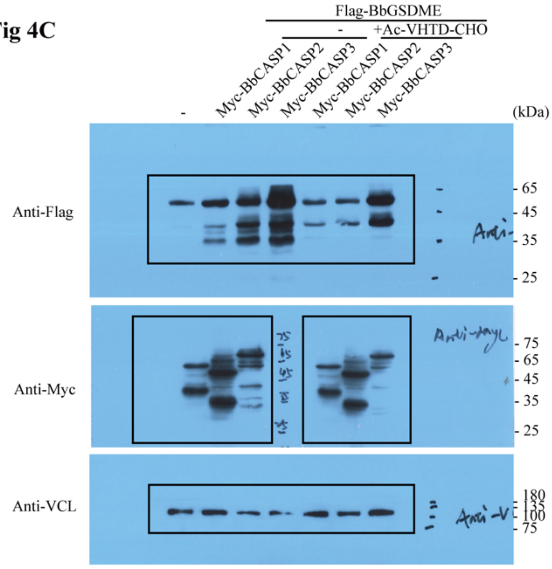

Fig 4D

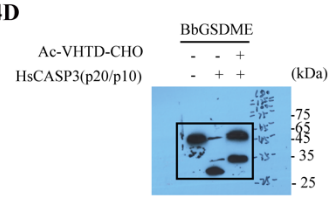

Fig 4E

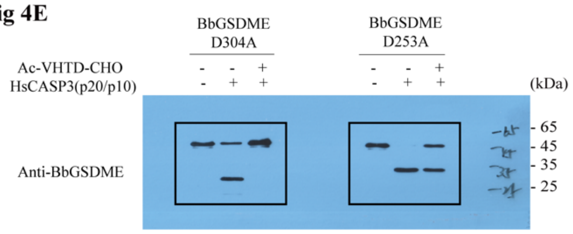

Fig 4I

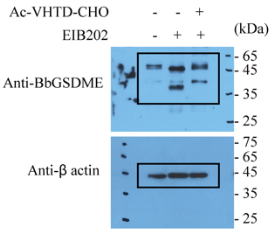

Fig 4O

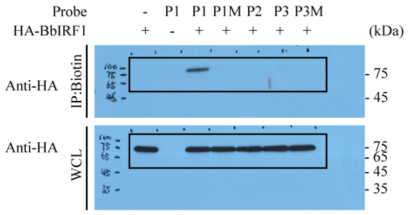

Fig 4P

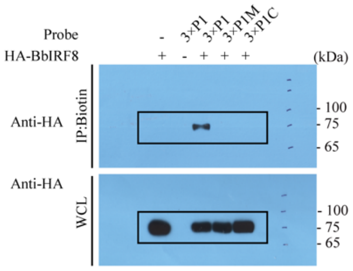

Fig 5

Fig5E

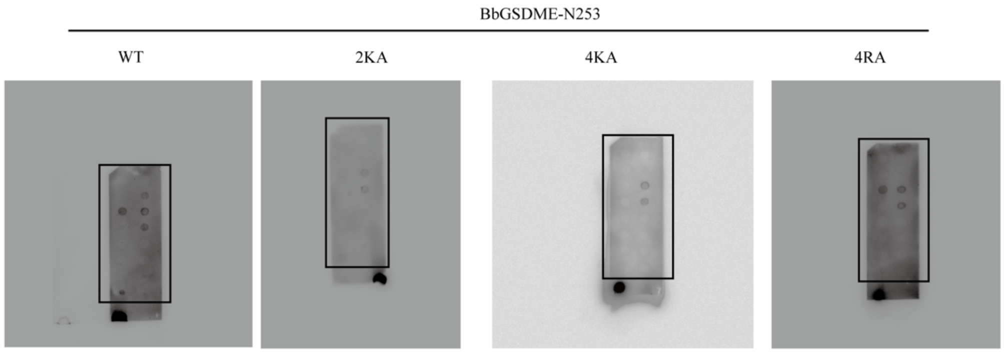

Fig5J

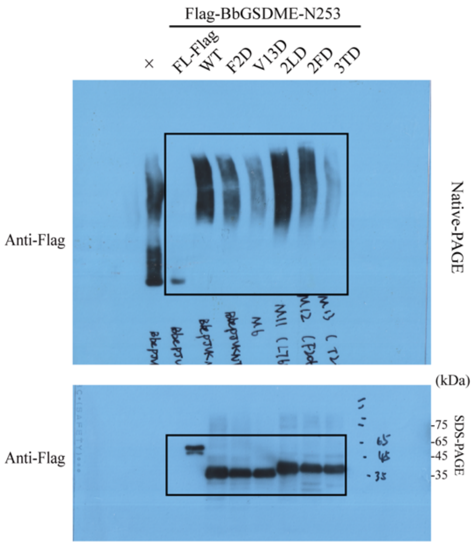

S4 Fig

S4C Fig

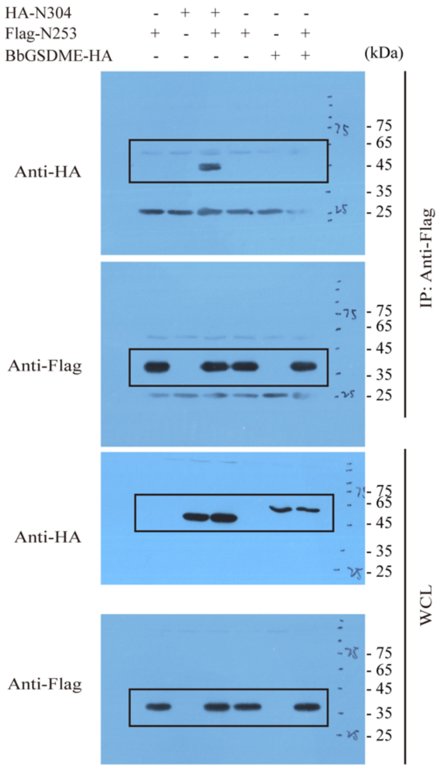

S4D Fig

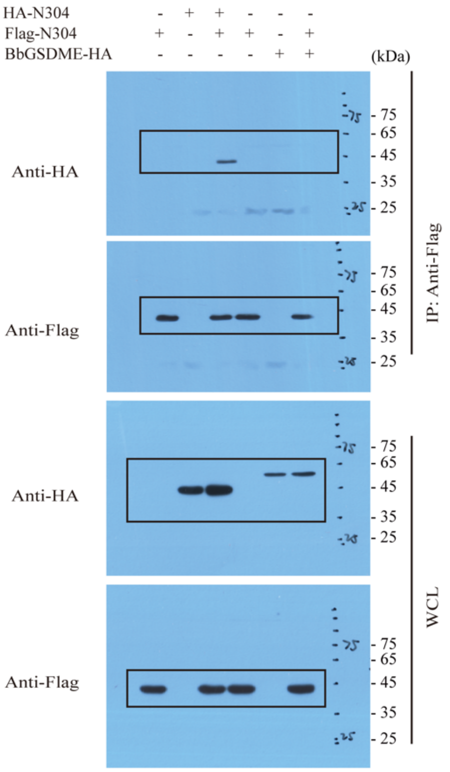

S4E Fig

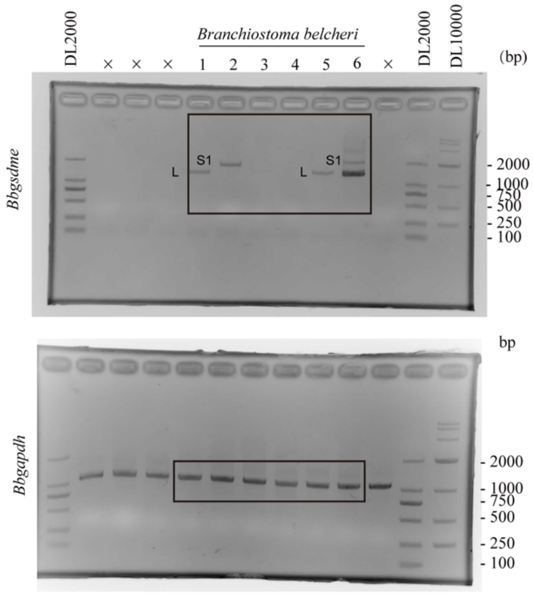

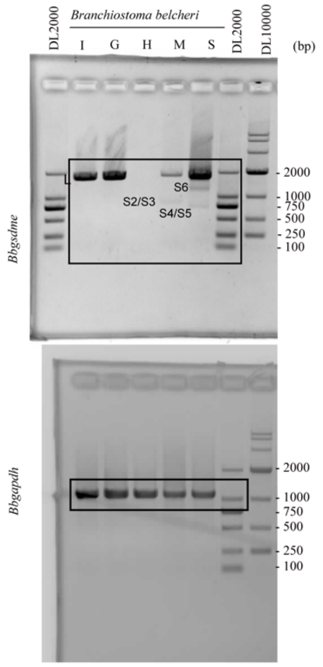

S6 Fig

S6D Fig

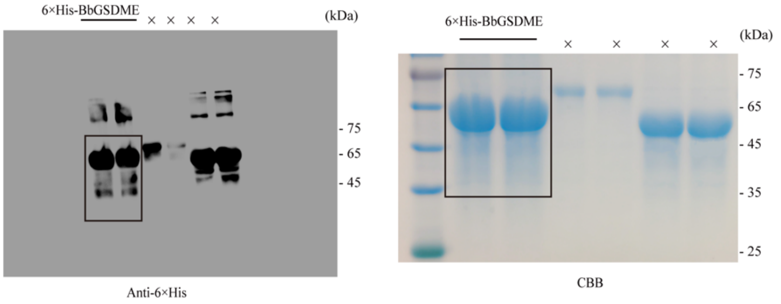

S6E Fig

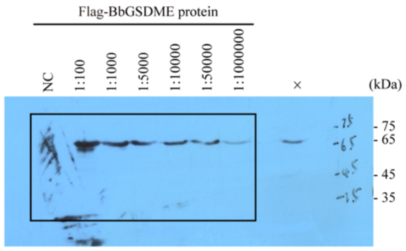

S6F Fig

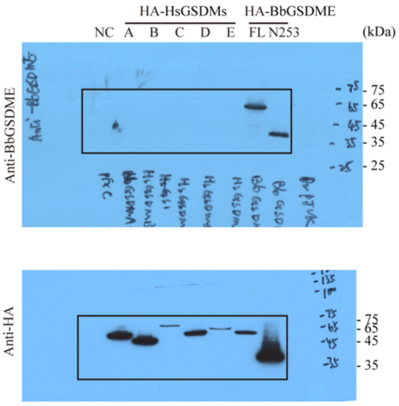

S6I Fig

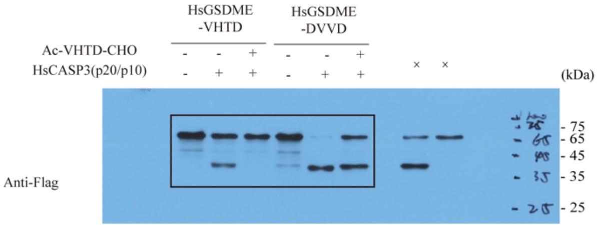

S8 Fig

S8B Fig

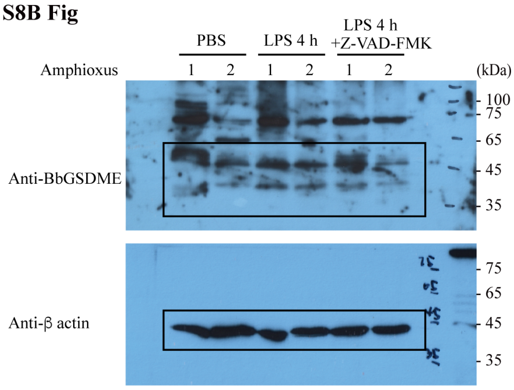

S8C Fig

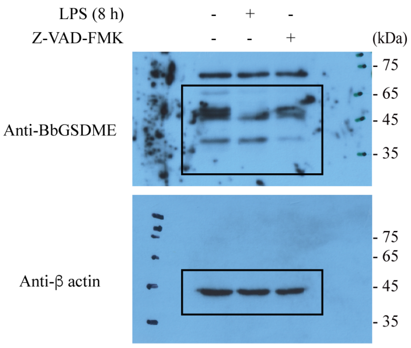

Supplement: S1 Raw Images — (PDF) [file pbio.3002062.s016.pdf]
